# Gene expression reversal toward pre-adult levels in the aging human brain and age-related loss of cellular identity

**DOI:** 10.1038/s41598-017-05927-4

**Published:** 2017-07-19

**Authors:** Handan Melike Dönertaş, Hamit İzgi, Altuğ Kamacıoğlu, Zhisong He, Philipp Khaitovich, Mehmet Somel

**Affiliations:** 1 Department of Biological Sciences, Middle East Technical University, 06800 Ankara, Turkey; 2 Department of Molecular Biology and Genetics, Bilkent University, Ankara, Turkey; 3 CAS Key Laboratory of Computational Biology, CAS-MPG Partner Institute for Computational Biology, 320 Yue Yang Road, Shanghai, 200031 China; 4 Max Planck Institute for Evolutionary Anthropology, Deutscher Platz 6, Leipzig, 04103 Germany; 5 European Molecular Biology Laboratory, European Bioinformatics Institute, Wellcome Trust Genome Campus, Hinxton, Cambridge CB10 1SD United Kingdom

## Abstract

It was previously reported that mRNA expression levels in the prefrontal cortex at old age start to resemble pre-adult levels. Such expression reversals could imply loss of cellular identity in the aging brain, and provide a link between aging-related molecular changes and functional decline. Here we analyzed 19 brain transcriptome age-series datasets, comprising 17 diverse brain regions, to investigate the ubiquity and functional properties of expression reversal in the human brain. Across all 19 datasets, 25 genes were consistently up-regulated during postnatal development and down-regulated in aging, displaying an “up-down” pattern that was significant as determined by random permutations. In addition, 113 biological processes, including neuronal and synaptic functions, were consistently associated with genes showing an up-down tendency among all datasets. Genes up-regulated during *in vitro* neuronal differentiation also displayed a tendency for up-down reversal, although at levels comparable to other genes. We argue that reversals may not represent aging-related neuronal loss. Instead, expression reversals may be associated with aging-related accumulation of stochastic effects that lead to loss of functional and structural identity in neurons.

## Introduction

The human brain undergoes significant structural and functional changes during both prenatal and postnatal development^1, 2^. However, changes are not limited to the developmental period, but continue in adult individuals over their lifetime, termed brain aging^3^. Aging-related changes include loss of gray and white matter volume, increased inflammation, loss of dendritic spines, elevated axonal bouton turnover rates, and a general loss of synaptic plasticity, which is paralleled by declining cognitive function and elevated risk of neurodegenerative disease^4–7^. Nevertheless, the molecular mechanisms behind aging-related phenotypic changes are only barely understood.

One method to study molecular mechanisms of aging is transcriptome analysis, *i.e*. investigating how mRNA levels change with adult age. A prefrontal cortex transcriptome age-series analysis we had conducted earlier found that the majority of gene expression changes in aging (post-20 years of age) occurred in the opposite direction of gene expression changes in postnatal development (pre-20 years of age)^8^. More specifically, genes up- (or down-) regulated during postnatal development were down- (or up-) regulated in aging, displaying a “reversal” pattern. A similar observation was made in a subsequent, independent study of frontal cortex aging^9^, suggesting that reversal in brain aging may be a common trend.

Processes important for the establishment of functional neuronal networks, such as axonogenesis, myelination, synaptogenesis and synaptic maturation, are active during postnatal development^10, 11^ and involve specific gene expression changes in neurons^8, 9, 12^. These molecular and morphological changes are thought to help constitute the cellular identity of neurons, that is, the molecular and physiological characteristics that define a mature, differentiated cell. Aging-related expression reversal among genes involved in these processes may represent the loss of cellular identity and/or declining physiological activity in old neurons, and a possible link to functional cognitive decline with age.

The prevalence of gene expression reversal in aging has not yet been systematically tested across different brain regions or in different datasets. Nor has it been shown that reversal is associated with neuronal functions, as would be expected under the loss of cellular identity model. Here we use multiple datasets that measured transcriptome profiles across postnatal development and aging in diverse brain regions, to gain insight into the prevalence and biological essence of the reversal trend. We confirm the presence of a common gene set showing reversal in their expression profiles across different brain regions, in the form of developmental up-regulation followed by down-regulation in aging (“up-down”). We further study the functional associations of these expression patterns.

## Results

In order to compare molecular changes in the brain before and after adulthood, we compiled data from three published human postmortem brain transcriptome age-series covering postnatal lifetime, produced by three different laboratories, each based on different microarray platforms. To our knowledge, these are the only brain transcriptome datasets that include samples distributed across the whole human postnatal lifetime. The 19 datasets comprise 17 brain regions and 1,017 samples in total, and individual ages span from 0 to 98 years of age (Table 1, Figure S1).

**Table 1:** Datasets used in the study. The brain region abbreviations stand for: A1C: Primary Auditory Cortex; AMY: Amygdala; CBC: Cerebellar Cortex; DFC: Dorsolateral Prefrontal Cortex; HIP: Hippocampus; IPC: Posterior Inferior Parietal Cortex; ITC: Inferior Temporal Cortex; M1C: Primary Motor Cortex; MD: Mediodorsal Nucleus of the Thalamus; MFC: Medial Prefrontal Cortex; OFC: Orbital Prefrontal Cortex; PFC: Prefrontal Cortex; S1C: Primary Somatosensory Cortex; STC: Superior Temporal Cortex; STR: Striatum; V1C: Primary Visual Cortex; VFC: Ventrolateral Prefrontal Cortex.

GEO Accession	Data source Name	Brain regions	Number of samples*	Age distribution	Microarray platform
GSE18069 GSE22569	Somel2011	PFC, CBC	45 (25)	0–98	HuGene-1_0-st
GSE25219	Kang2011	A1C, AMY, CBC, DFC, HIP, IPC, ITC, M1C, MD, MFC, OFC, S1C, STC, STR, V1C, VFC	741 (33)	0–82	HuEx-1_0-st
GSE30272	Colantuoni2011	PFC	231	0–78	Illumina Human 49 K Oligo array (HEEBO-7 set)

^*^Numbers in the parentheses show the number of individuals used in the study.

We divided each dataset in two parts by categorizing samples from individuals between 0–20 years of age as (postnatal) “development”, and those from individuals 20 years of age or older as “aging”. This division is based on the approximate age of first reproduction in diverse human societies^13^, and it also represents a general turning point in brain development and functionality, such as the initiation of certain aging-related cognitive decline trends^6^ (we confirm that using earlier or later turning points yields qualitatively similar results in downstream analyses; see Methods and Figure S13). Separating each dataset into development and aging yielded 38 subdatasets in total.

In downstream analyses, we model gene expression changes as linear processes (*i.e.* increase or decrease) in both periods, *i.e*. development and aging, separately. For this reason, we did not include prenatal brain samples in this study, as developmental changes can be discontinuous between pre- and postnatal periods at both histological^10, 11^ and molecular^9, 12^ levels.

### Gene expression change during development and aging

**Figure 1:**
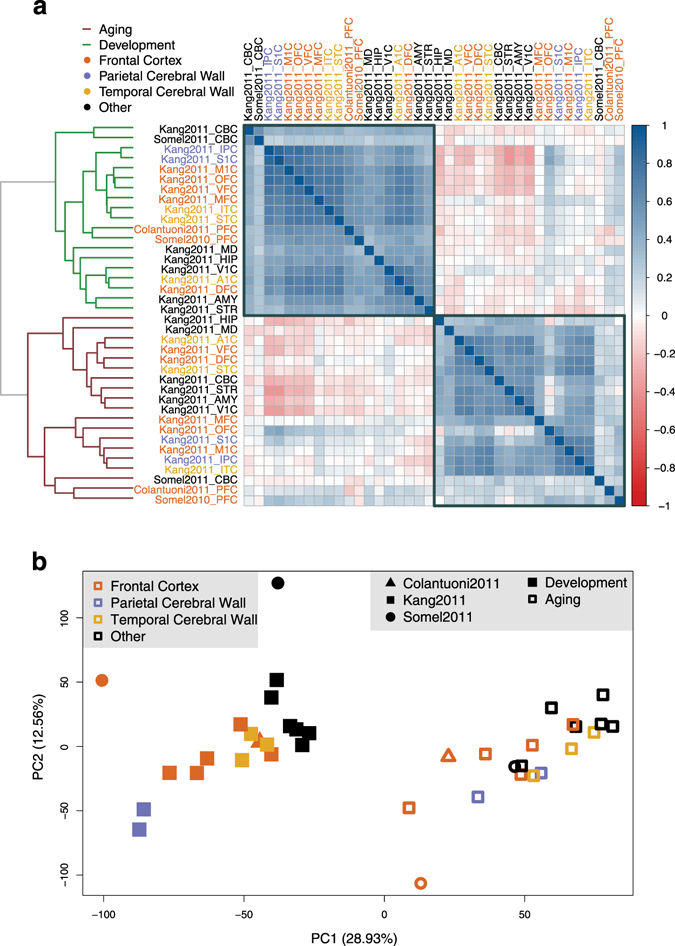
Expression changes during brain postnatal development and aging. (**a**) Correlations among expression-age correlation coefficients (in development or in aging) across subdatasets. The color of the squares changes with the magnitude of the Spearman correlation coefficient between pairs of subdatasets (across 11,563–22,713 common genes in each pairwise comparison); darker colors show stronger negative (red) or positive (blue) correlation. Row and column labels indicate the brain region and the edge color of the dendrogram shows the time period of datasets. The order of brain regions is determined by agglomerative hierarchical clustering using the Spearman correlation coefficients between datasets based on the age-related expression changes. (**b**) Principle components analysis of age-related expression change in brain regions. The analysis was conducted on an age-expression Spearman correlation coefficient matrix of 11,258 genes and 38 subdatasets. The x- and y-axes show the first and second principle components. The values in the parentheses show the variation explained by each component.

To obtain an overview of the data, we measured age-related change for each gene using the Spearman correlation coefficient (rho) between individual age and expression levels, separately for each subdataset (14,356–22,714 genes). We then studied the consistency of these age-related change measures in development and in aging across all subdatasets. Specifically, we calculated the correlation of age-expression correlations among all pairs of subdataset combinations. Within each period, different datasets show positive correlation with each other, indicating parallel age-related changes (Fig. 1a). However, higher correlation is observed among developmental datasets (median rho = 0.52) than in aging (median rho = 0.40) (Wilcoxon signed rank test p < 10^−15^), a result that may be related to higher gene expression noise in aging than in development (see Discussion).

We next conducted principle components analysis on age-related change trends measured in subdatasets. We observed that ontologically related brain regions cluster more visibly in development than in aging (Fig. 1b). This result again implies higher stochasticity in age-related expression changes during aging than in development.

Notably, both analyses revealed that transcriptome-wide age-related change trends are distinct between development and aging (Fig. 1). In fact, correlations between age-related expression change measured in development and in aging were frequently negative, in line with reversal of gene expression during aging to pre-adult levels (55% of pairwise comparisons were negative, as shown with reddish tone in Fig. 1a; median rho = −0.02).

### Shared age-related expression change across datasets

**Figure 2:**
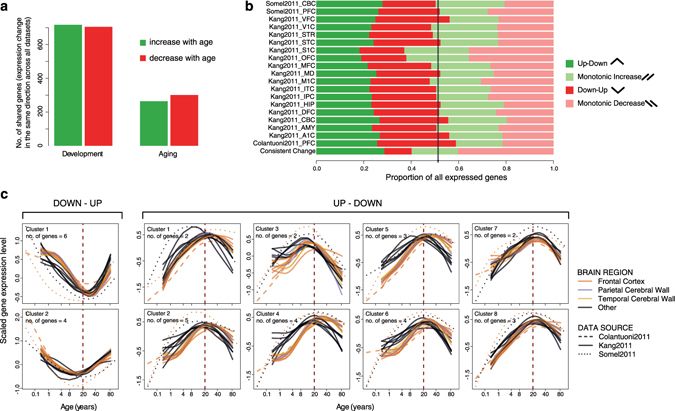
Shared expression changes with age and shared reversals. (**a**) The number of consistent expression changes in development and aging. Interestingly, there is a trend toward 1.7% more consistently up-regulated genes during development, and 12.3% more consistently down-regulated genes during aging. This was also in line with 16/19 datasets harboring more up-regulated significant genes during development, and 10/12 datasets harboring more down-regulated significant genes during aging (Figure S2). (**b**) The proportion of different trends in age-related expression change in each dataset and among the genes showing consistent change across datasets. No effect size or significance cutoff was used. Up-down: up-regulation in development and down-regulation in aging; Down-up: down-regulation in development and up-regulation in aging; Monotonic increase: up-regulation in development and up-regulation in aging; Monotonic decrease: down-regulation in development and down-regulation in aging. (**c**) Average expression trajectories of consistent reversal gene clusters, plotted against individual age. The x-axis shows the age of samples on the fourth root scale and the y-axis shows the scaled mean expression level for each cluster. The spline curves indicate the mean expression change with age for each dataset and brain region.

We next sought to identify shared age-related change patterns in development and in aging across the 19 datasets. For this, we first tested each gene for age-related expression change in each of the subdatasets separately, correcting for multiple testing. This revealed between 0–30% (median 2%) of genes showing significant change during development among datasets, and 0–9% (median 0%) of genes showing significant change during aging (Figure S2). Thus, in either development or in aging, no common genes can be detected with this approach, which likely reflects both biological and technical noise preventing age-related change from being reliably detected in a single dataset. We therefore resorted to an alternative approach, and asked if we could identify common development- or aging-related genes that show expression change in the same direction across all 19 datasets (Fig. 2a). Specifically, we categorized all genes as showing up- versus down-regulation trends with age, irrespective of effect size; we then identified the set of common genes showing the same trend in all datasets; and finally, we tested whether a set of such size can be expected randomly, using 1,000 structured permutations of individual labels in each dataset (i.e. randomizing age while keeping the individual identities fixed across all datasets from each data source; see Methods).

Using this approach, we found 1422 genes (13% of 11,258 genes expressed in all datasets) showing consistent expression change during development across the 19 datasets, while only 149 are expected by chance (one-sided permutation test p < 0.001; Figure S3a). For aging, we found 565 (5%) such consistent genes (expected = 156, one-sided p = 0.008; Figure S3b). Thus, although age-related change trends may be too weak to be detected in individual datasets, searching for consistency across datasets and testing the result against the null hypothesis of no shared age-related change can provide sufficient power to determine shared trends.

### Microarrays do not bias against identifying aging-related expression change

Why is there a deficiency of significant aging-related expression changes, relative to those in development? This is not simply related to differences in statistical power, as all datasets comprise more adults than pre-adults (Figure S1). Another explanation pertains to technical artifacts such as the use of microarrays. Microarrays cannot measure absolute expression levels as accurately as RNA-sequencing (RNA-seq)^14^ and they only probe a pre-determined set of genes and isoforms. But because of a lack of well-powered RNA-seq studies that include samples representing the whole lifespan (see Methods), our study was restricted to three microarray experiments. Could microarray data be biased against identification of aging-related expression change? To address this we calculated expression-age correlations using an RNA-seq dataset including 13 different brain regions produced by the GTEx Consortium^15^ and compared these with expression-age correlations calculated using the microarray-based datasets. This showed no indication of clustering by platform (Figure S4). We also examined the possibility of a gene representation bias in microarray studies. Comparing expression-age correlations among all genes detected in an RNA-seq dataset with the ones detected by both RNA-seq and microarray datasets also revealed no indication that genes represented on the microarrays are biased towards higher or lower age-expression correlation with age (Figure S5). We conclude that use of microarray data is likely not the cause of the low numbers of aging-related genes identified.

### Shared reversal trends across datasets

We next studied reversal, *i.e*. a gene’s expression levels changing in the opposite direction between development and aging. Because in most datasets no genes show significant expression change during aging, instead of limiting our analysis to the few individually significant genes, we used the advantage of the multiple datasets available and sought shared *trends* across all 19 datasets (irrespective of effect size of age-related change, as above). For this, we first classified all expressed genes into 4 categories, depending on their up- or down-regulation tendencies in development or in aging, in each dataset (Fig. 2b). This revealed ~50% (37–59%) of genes showing reversal trends across the 19 datasets (notably, a 50% ratio would already be expected by chance). Next, we used two approaches to test the statistical significance of the observed reversal trends.

In the first approach, we determined the set of genes showing shared reversal trends across all 19 datasets (again irrespective of effect size) and calculated the significance of this gene set using random permutations of individual labels in each dataset (see Methods). There were 87 genes showing consistent change across all datasets both in development and in aging. Among these, 35 showed consistent reversal (Fig. 2c and Table S1). Of these 25 showed an up-down pattern (up-regulation in development and down-regulation in aging) shared across all 19 datasets, which was significantly more than by chance (expected = 5, one-sided p = 0.031). Meanwhile, 10 showed a down-up pattern, which was non-significant (expected = 5, one-sided p = 0.246). We confirmed the up-down trends identified among the 25 genes in additional transcriptome datasets of brain development and aging (permutation test p < 0.0001; see Methods).

### Shared functional processes enriched in reversal trends

**Figure 3:**
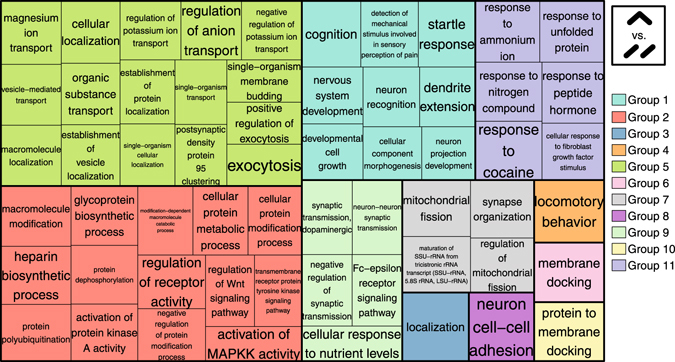
Shared GO Biological Process (BP) categories enriched for up-down genes. The 113 GO BP categories were chosen for showing a trend for enrichment in up-down genes (odds ratio > 1) in all 19 datasets (p = 0.017). The 113 categories were summarized by REVIGO^16^. Different colors show different superclusters and the size of each box is determined by the uniqueness of the categories.

In the second approach, we tested whether particular functional categories (not necessarily individual genes) show shared enrichment in reversal patterns across all the 19 datasets. Here we compared the reversal proportion among genes assigned to a Gene Ontology (GO) category with the reversal proportion among all other genes, thus calculating an odds ratio for reversal (see Methods and Figure S6a). Importantly, we kept the developmental trend constant, such that we compared the up-down (or down-up) pattern, with the up-up (or down-down) pattern. In total, we calculated reversal odds ratios for 13,392 GO Biological Process (BP) categories. We found 11 shared categories with more down-up than down-down genes across all 19 datasets (odds ratio >1), which involves categories related to differentiation and morphogenesis (Table S2); but the result was not significant in permutations of individual ages (p = 0.4). In contrast, there were 113 shared categories with more up-down than up-up genes in all datasets, more than expected by chance (expected = 11, one-sided p = 0.017, Figure S3c). Categories enriched in up-down genes vs. up-up genes were mainly involved in neuronal functions, synaptic functions, diverse macromolecule modification and localization, as well as signaling processes (Fig. 3 and Table S3). We also repeated the analysis after removal of duplicated GO BP categories and showed that the significance of shared categories was not driven by the presence of duplicated categories (Figure S7).

When we repeated the same analysis for GO Molecular Function (MF) and GO Cellular Component (CC) categories, we did not detect significantly shared categories for the down-up pattern across different datasets (Table S4 and Table S6), whereas categories enriched in up-down pattern were found to be significant (one-sided p = 0.037 for GO MF and p = 0.008 for GO CC). Shared up-down GO MF categories are mainly related to post-translational modifications (Table S5) and shared up-down GO CC categories involve mainly neuronal or synapse-related genes (Table S7).

Thus, both analyses showed that one reversal pattern, up-down, can be consistently detected in brain aging across diverse brain regions, and is significantly associated with specific functional categories, including certain neural functions. In the following analyses, we investigate the biological significance of up-down expression patterns to test: (1) whether up-down reversal may be driven by *trans* regulators, (2) whether up-down reversal represents neuronal loss, (3) whether up-down reversal involves genes with roles in neuronal differentiation, which would be compatible with the loss of cellular identity hypothesis, and (4) whether up-down reversal shows association with genes dysregulated in Alzheimer’s Disease.

### Shared regulators of up-down reversal trends

We asked whether genes showing up-down patterns may be regulated by specific *trans* factors. To investigate this we tested whether up-down genes, compared to up-up genes, were enriched among targets of specific microRNAs (miRNAs) or transcription factors (TFs) (see Methods). Testing 1078 miRNAs and 211 TF binding sites separately, we found one miRNA and 4 TFs consistently enriched across the 19 datasets for the up-down pattern, relative to up-up; however, the results were non-significant and only marginally significant compared to permutations (one-sided p = 0.56 and p = 0.069), respectively.

### Altered neuronal contribution during aging

Given that neuronal processes up-regulated during postnatal development show decreasing expression during aging across all brain regions, we first checked whether genes assigned to neuron-related categories *overall* show more up-down reversal compared to non-neuronal genes. A shared trend was observed which was marginally significant (Figure S8, one-sided p = 0.095), implying that up-down reversal is likely not specific to neuronal genes. We further asked whether neuronal expression might show overall reduction relative to those of other CNS cell types. To address this we used two more published datasets: a mouse brain cell type-specific microarray dataset^17^, and a human brain single cell RNA-sequencing dataset^18^. We then briefly studied the relative contributions of different cell types’ transcriptomes (including neurons, astrocytes, oligodendrocytes) to each sample in the age-series datasets, using a simple linear regression-based deconvolution approach (Methods). In all datasets expression levels in whole tissue predominantly reflect neuronal expression throughout the lifespan﻿,﻿ as opposed to expression﻿ from other cell types﻿ (Figure S9). A subtle decrease in neuronal contribution was detectable across most datasets: Depending on the cell type specific dataset we used, 90–100% of datasets showed decreasing relative neuronal expression during aging (Figure S9; the median correlation coefficient between age and neuronal contribution across datasets ranged between −0.10 to −0.35). A parallel, consistent increase in astrocyte contributions could also be observed. We note, however, that because this analysis relies on expression profile comparisons from different platforms, the result cannot be directly interpreted as altered cell type proportions. Cell autonomous but systemic loss of gene expression in neurons can also lower neuronal contribution to the tissue level mRNA pool (see Discussion).

### Up-down reversal and neuronal differentiation

**Figure 4:**
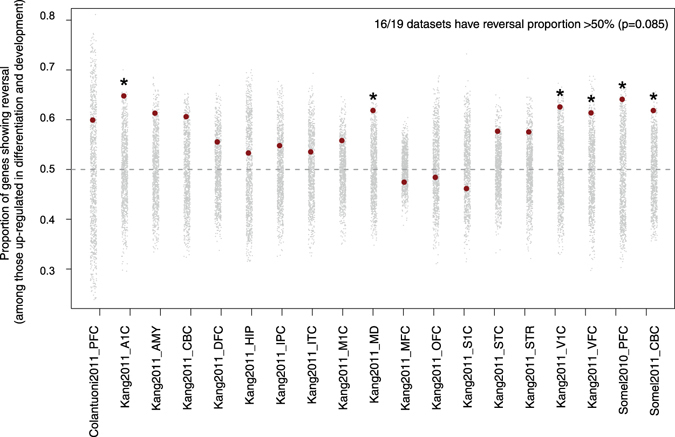
Reversal proportion of the genes up-regulated during *in vitro* neuronal differentiation and in postnatal development. The gray points show the reversal proportion in 1000 permutations of individual labels in each dataset, whereas the red points show the observed proportions (*i.e*. number of up-down genes divided by the number of up-up genes, among genes up-regulated during neuronal differentiation). Statistical significance of each dataset is indicated by asterisks, which represent nominal p-values from permutations of individual ages (without correction for multiple testing).

To test a possible association between reversal in aging and loss of cellular identity, we compared reversal patterns with expression patterns related to differentiation. Our first hypothesis was that differentiation-related genes should show more up-down patterns than up-up patterns. For this, we used a human iPSC-derived neuronal differentiation dataset^19^. We determined that 476 to 651 genes showed up-regulation both during *in vitro* differentiation and during development across the 19 datasets (see Methods); these are ideal candidates for having role in postnatal neuronal identity establishment. We found that these genes are prone to show reversal, *i.e*. be down-regulated during aging, relative to up-regulation in aging: 16/19 datasets showed more up-down reversal than up-up patterns (Fig. 4), whereas no significant trend was observed in the opposite direction, significance being measured by permutations of individual ages in aging datasets. Still, the overall significance of finding 16/19 datasets was only marginal (one-sided p = 0.085). Second, we tested whether differentiation related genes show more up-down patterns than non-differentiation related genes by permuting sample stages in the iPSC-derived neuronal differentiation dataset, which was not significant (one-sided p = 0.144). Thus, genes related to differentiation and that are up-regulated during postnatal development are also inclined to be down-regulated during aging, in line with the notion of cellular identity loss. Meanwhile the up-down trend among differentiation-related genes is comparable to the rest of the transcriptome, not necessarily stronger.

### Up-down reversal and Alzheimer’s Disease

If cellular identity loss is genuine, it may also characterize aging-related neurodegenerative disease. This led us to ask whether up-down reversal might be paralleled by expression changes observed under conditions such as Alzheimer’s Disease (AD). Combining 7 datasets of AD involving 6 different brain regions, we determined 690 and 653 genes consistently up- and down-regulated in AD versus age-matched healthy controls across all AD datasets, respectively (see Methods). We first confirmed that expression changes are largely parallel between AD and aging, as shown earlier^20^ (Figure S10; see Methods). We next studied whether genes up-regulated in development (relative to genes down-regulated in development) are frequently also down-regulated in AD, where we found marginally significant trends (one-sided p = 0.092 and p = 0.052 by permuting development or AD datasets, respectively). We further asked whether genes down-regulated in AD tend to show up-down reversal more often than the down-down pattern (in order to control for background similarity between AD and aging). This trend was present in 14/19 datasets but was non-significant when testing by permuting development datasets (one-sided p = 0.4). Thus, AD-related expression down regulation overlaps with, although is not exclusively associated with, up-down reversals.

## Discussion

Aging, unlike development, is frequently considered *not* to be an evolutionarily programmed process that is adaptive *per se*, but a result of stochastic evolutionary and cellular/physiological events^21^. Likewise, aging-related molecular changes are supposed to be driven by accumulating stochastic events, affecting each individual, and possibly each cell, differently. As a result, studying aging phenotypes with limited sample sizes is challenging. This is especially so in humans, where the environment is uncontrolled.

Here we adopted a meta-analysis approach that, instead of seeking for significant signals in individual datasets, focuses on shared *tendencies* among multiple distinct datasets and different brain regions. Our approach will miss region-specific aging patterns. But at the same time, it has high sensitivity for shared expression change trends, because it includes trends too weak to pass significance thresholds in a single dataset. Our method is expected to minimize the influence of the confounding factors in individual datasets and thereby improve specificity and reproducibility. The approach can further reveal shared trends at the functional category level instead of the single gene level.

Our analysis identified shared and statistically significant expression change patterns in brain development and in brain aging, leading to a number of observations:

### Noise in aging

We found conspicuously more shared expression change during development than in aging (Figs 1 and 2a), which was not a statistical power issue (Figure S1), nor a technical artifact (Figures S4 and S5). Rather, weaker expression changes in aging could occur because developmental expression changes are of higher magnitude (hence with higher signal/noise ratio) than those in aging^8^. Alternatively, aging-related expression changes may involve higher inter- and intra-individual variability than in development^9, 22, 23^, which could arise due to stochastic environmental or cellular effects. We also found that down-regulations were more prominent among shared aging-related changes than among development-related changes (Fig. 2a), again implying that aging-related expression changes may be particularly influenced by disruptive stochastic effects which may be expected to drive down-regulation more frequently than up-regulation. Thus, shared expression patterns across datasets hint at aging being subject to higher noise than development.

### Prevalence of up-down patterns

We identified shared up-down reversal patterns across the 19 different datasets, involving 25 genes (shared down-up patterns were not significant). More importantly we found significant similarities among datasets at the pathway level: genes showing up-down trends were enriched among specific functional categories including neuron-related and synaptic processes. Genes activated in iPSC-derived neuronal differentiation and in postnatal development, which could include genes critical for neuronal function, also tended to be down-regulated during aging (even though at levels comparable to other gene sets). Combined, these results suggest that genes associated with neuronal and synaptic function, among others, may lose activity with advancing age in the brain, reminiscent of aging-related synaptic loss in the mammalian brain, a major culprit for aging-related decline in cognitive abilities^24^.

Why would critical neuronal genes be down-regulated during aging, rather than maintain their young adult expression levels? Here we consider three hypotheses: (a) extension of synaptic pruning, (b) cell type composition changes, and (c) cellular identity loss caused by damage and epigenetic mutations.

### Extension of synaptic pruning

**Figure 5:**
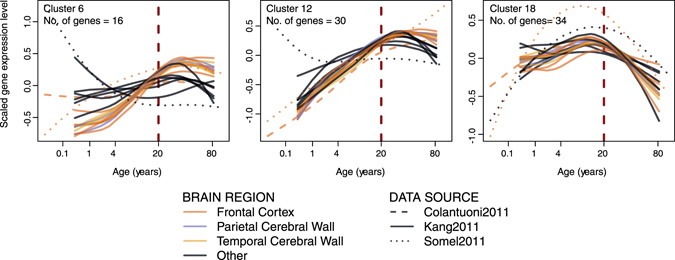
Three examples of expression-age trajectories among clusters of synapse-related and up-down-enriched GO BP categories (Group 9 in Fig. 3). The y-axis shows the scaled expression level and x-axis shows the age of samples on the fourth root scale. The spline curves indicate the mean expression change with age among cluster member genes for each dataset and brain region (for all of the clusters see Figure S11). The three clusters were chosen for showing peak expression at ages earlier or later than 20 years of age.

One possible culprit behind the up-down reversal pattern is synaptic pruning, a developmental process that initiates in postnatal development and may drive down-regulation of synaptic genes after childhood^25^. If synaptic pruning is indeed responsible for the observed up-down patterns, we expect up-regulation trends to be replaced by down-regulation already during childhood. In other words we would expect gene expression turning points arising before 20 years of age. Clustering all 25 shared up-down genes’ expression patterns and inspecting their turning points (*i.e*. maxima), we found that most clusters, although not all, had turning points around 20 years of age (Fig. 2c). Repeating this clustering analysis with 638 genes in all synapse-related categories (categories clustered as “Group 9” in Fig. 3), we found a range of turning points supported by multiple datasets (Figure S11). Specifically, while some gene clusters peak early in life (see Cluster 18 in Fig. 5), others clearly peak after 20 years of age (see Clusters 6 and 12 in Fig. 5). Overall, most up-down patterns do not arise before adulthood, arguing against the possibility that reversals represent the continuation of developmental processes that initiate during childhood.

### Cell type composition change

The brain is a heterogeneous tissue comprised of different cell types, whereas all the analyzed datasets have been produced using whole tissue samples. This raises the question whether the up-down reversal pattern associated with neuronal processes represents cell autonomous gene expression changes, or changes in brain cell type proportions^26^.

In contrast to the established phenomenon of synaptic loss with age^5, 27^, histological evidence for aging-related cell type composition change, specifically neuronal loss, remains unclear. Multiple stereological studies of the primate cortex have reported no aging-related neuronal loss^28, 29^. Meanwhile, a fractionation experiment in the rat brain found ~30% decrease in neuron numbers between adolescence and old age^30^ (but the method remains to be applied to primate brain samples). Finally, a recent study using image analysis of NeuN-marked human brain sections reported loss of only large bodied neurons, representing 20% of the neuron population^31^. Our deconvolution analysis results are also equivocal (Figure S9): they could be compatible with modest neuronal loss during human brain aging, but also with cell autonomous loss of neuron-specific expression.

If neuronal loss was the main source of shared up-down patterns among brain regions, we may expect coordinated expression changes that affect multiple neuron-specific markers shared among brain regions. These would also be expected to be shared at the single gene level. However, the up-down reversal patterns are mainly shared at the functional process level, rather than at the gene level. This argues against a major role for cell type composition shifts driving up-down reversal. Nevertheless, shared neuronal loss remains a possibility if low signal/noise ratios could be blurring putative shared expression patterns. The question hence awaits to be addressed by future cell type-specific age-series datasets that cover the whole lifetime.

### Loss of cellular identity

Finally, shared up-down expression patterns could also be driven by age-related cellular damage and genetic/epigenetic mutation accumulation. We can postulate two mechanisms: (a) the accumulation of stochastic mutations or epimutations directly disrupting normal neuronal function, or (b) regulated survival responses under accumulating insults^32^.

In this regard, it is interesting that we cannot definitely associate up-down reversal patterns with common regulators such as miRNA and TFs. If not a false negative result, this may suggest alternative regulatory factors (*e.g*. chromatin modifiers, RNA binding proteins, or yet unidentified TFs) driving up-down patterns. But it may also suggest that up-down reversal is driven by stochastic effects, such as accumulating DNA or protein damage or epigenetic mutations. If such stochastic effects can convergently disrupt the expression of a common, vulnerable set of neuronal genes across multiple cells and across different individuals, they could give rise to shared up-down reversal patterns. Future single cell RNA-sequencing and epigenomics age-series that include both development and aging may help illuminate the exact drivers of up-down reversal.

We find that genes prone to down-regulation in AD do not show exclusive up-down patterns in normal subjects. This limited overlap between AD and up-down reversal may be expected, as most expression down-regulation in AD is likely driven by acute processes such as neuronal apoptosis^33^, whereas up-down patterns in normal aging probably involve more subtle aging phenotypes, such as synaptic loss.

In summary, the up-down reversal phenomenon supports the notion that synaptic loss and cognitive decline observed in normal brain aging may be linked to gradual cellular identity loss, driven by accumulating stochastic intracellular events.

## Methods

### Data retrieval and pre-processing

#### Choice of age-series datasets

All data analyzed in this study correspond to human brain gene expression profiles measured through microarray experiments. To our knowledge, there exists only one RNA-sequencing dataset that covers the whole lifespan, published by Mazin *et al* ^34^., but this dataset is underpowered and its subjects are also included in the Somel2011^8, 35^ dataset; we therefore chose not to include this RNA-sequencing dataset in our study. We used 3 different data sources spanning 17 different brain regions –not mutually exclusive- and including the whole postnatal lifespan^8, 9, 12, 35^. We did not include prenatal samples present in two of these datasets. The reason for this is related to the observation that expression change dynamics in prenatal and postnatal periods are tend to be discontinuous (see refs 9, 12). This is expected, given that major processes e.g. neurogenesis mainly happen in the fetal period, whereas others e.g. myelination are mainly postnatal^10, 11^. Because our motivation is to understand the molecular dynamics of brain aging in relation to changes occurring before adulthood, here we limit ourselves to comparing aging with postnatal development. Finally, we did not attempt to combine independent development and aging datasets (e.g. RNA-seq datasets) to study reversal, because this could confound technical and biological effects. Still, we mark the need for well-powered RNA-sequencing studies of gene and isoform expression changes during brain development and aging that will allow better understanding of molecular aging dynamics. ***Preprocessing age-series datasets:*** The combined pool of individuals’ age range spans 0 to 98 years of age (Figure S1). The data was accessed through the NCBI Gene Expression Omnibus^36^ (GEO) Accession numbers provided in Table 1 ^37^. Gene expression datasets from two data sources, Somel2011 and Kang2011, were processed by RMA correction (using “oligo” library^38^ in R), log2 transformation and quantile normalization (using the “preprocess Core” library^39^ in R). Gene annotations for the probe-sets in Somel2011 data were obtained from the Ensembl database (v84)^40^ through the “biomaRt” R library^41, 42^. The annotations for the data source Kang2011 (for which probe-set IDs are not fully listed in Ensembl) was obtained from the GPL file deposited in GEO. The Colantuoni2011 dataset was Illumina-based and no public R packages are available for analyzing the raw data. We thus used the data processed by the study’s authors. Gene annotations for this data source were also retrieved using the GEO GPL file, and the Entrez Gene IDs were converted to the corresponding Ensembl (v84) gene IDs using the “biomaRt” R library. By visual inspection of the first and second principle components of the probe-set expression levels, outliers with the accession numbers GSM705108, GSM705202 GSM704438, GSM704567, GSM704226, GSM704227, GSM704627 (Kang2011) were excluded from the further analysis. ***Alzheimer’s Disease datasets:*** Seven different data sources^43–49^ were used for determining AD-related genes (Table S8). Preprocessing of the Blalock2004 dataset was performed using the R “affy” library^50^; and the “oligo” library was used for preprocessing the Hokama2014, Tan2010, and Antonell2013 datasets (following the suggestion of the package authors). For the other three datasets (not Affymetrix-based), we used series matrices deposited in GEO. According to the PCA results, two samples - GSM21205, GSM21207 (Blalock2004) - are detected as outliers by visual inspection. Three young samples (22 years old from Narayanan2014, 22 and 25 years old from Zhang2013) were removed from the analysis in order to keep the balance between the ages of the AD and ND samples. For the retrieval of Ensembl gene IDs for Blalock2004, we used Ensembl (v84) accessed through the “biomaRt” R package. For the other datasets (for which probe-set IDs are not fully listed in Ensembl), the GEO GPL file were used. For Miller2013, Narayanan2014, and Zhang2013 datasets, probe-set IDs are first converted to Entrez Gene IDs and then converted to Ensembl (v84) gene IDs using the “biomaRt” R package. ***Neuronal differentiation dataset:*** Gene expression levels corresponding to neuronal differentiation were downloaded from GEO database (GSE25542)^19^. The series matrix deposited in the database, which contains log2 transformed, quantile normalized probe-set expression levels, was used for the analysis. We first mapped probe-set IDs to Entrez Gene IDs using the GEO GPL file, and then converted these into corresponding Ensembl (v84) gene IDs using the “biomaRt” R package. ***Mouse cell type specific expression dataset:*** Contribution of different cell types to the transcriptome of each sample in the age-series datasets were analyzed using data downloaded from GEO database (GSE9566)^17^. GSE9566 contains expression profiles for different cell types in mouse brain, purified using FACS. Data preprocessing steps are the same as the other microarray datasets. Only the genes having 1-to-1 human orthologs are used for cell type-specific expression analysis. ***Summarizing probe-sets per gene*** *.* If a probe-set corresponded to multiple genes, it was removed from the dataset to ensure expression data was not duplicated. Expression data for genes having more than one probe-set annotation was summarized by calculating the mean expression level of the corresponding probe-sets, for each individual sample. ***RNA-seq dataset:*** We analyzed 13 different brain region transcriptome data generated by the GTEx project (v6p)^15^. We excluded genes having mean RPKM <1. The RPKM values provided in the GTEx database, log2 transformed and quantile normalized expression levels, are used for the downstream analysis. Similar to the microarray data, we excluded the outliers based on the visual inspection of the first and second principle components analysis. Distribution of the ages after outlier exclusion is given in Figure S12. ***scRNA-seq dataset:*** The single cell RNA-seq data of human brains with cell type information was retrieved from SRA (SRP057196)^18^. All the reads were mapped to the human genome hg38 with STAR 2.3.0e using the default parameters^51^. The number of reads covering the exonic regions of each protein-coding gene annotated in GENCODE^52^ v21 was counted and normalized using DESeq2^53^. Gene average expression levels for each cell type, represented as the number of fragments per kilobase per million reads (FPKM), were calculated as the mean FPKM across all cells marking as the cell type. Mean FPKM values are then log2 transformed and quantile normalized for the downstream analysis.

### Development vs. aging

To identify and compare expression changes in postnatal development and in aging, we divided individuals in each dataset using 20 years of age as point of separation (or turning point). In human societies, this corresponds to the age at first reproduction^13^. Earlier transcriptome studies^8, 9^ have also suggested age of 20 as a global turning point in brain gene expression trajectories. Nevertheless, in order to assess prevalence of reversal throughout the lifespan, the full analysis was repeated with different ages used as turning points (see *“different turning points”* and Figure S13).

### Age-related expression change

We used the Spearman correlation coefficient to assess age-related expression changes. P-values were corrected for multiple testing through the Benjamini-Yekutieli (BY) procedure^54^ using the “p.adjust” function in the base R library.

### Permutation test

We used random permutations of individual labels to assess the probability of finding the same or higher number of shared observations (*e.g*. number of shared genes across all datasets showing the same expression change pattern, or the number of shared GO groups across all datasets with odds ratio >1), and to estimate the false discovery rate (Figure S6b). We designed the permutation procedure to account for non-independence among subdatasets caused by the presence of the same subjects within Kang2011 and within Somel2011. Specifically, in each permutation, the individuals’ ages were randomly permuted within each data source and period (i.e. individual labels are permuted within development or aging samples to simulate the null hypothesis of no age effect within that period), and in each permutation, the same individuals were assigned the same age across different brain regions. We thus simulated the null hypothesis of no change during aging across the transcriptome, while maintaining dependence among genes and dependence among subdatasets. For permutations, we used the “sample” function in R.

### Gene expression clustering

In order to cluster genes according to their expression profiles across all datasets, the k-means algorithm (using the “kmeans” function in R) was used. We first standardized each gene’s expression level to mean = 0 and s.d. = 1. Directly combining scaled expression datasets and applying k-means would be misleading, because datasets have different number of samples. We therefore used standardized expression levels to calculate expression-age spline curves for each gene in each dataset (using the “smooth.spline” function in R) and interpolate at 20 equally distant age points within each dataset. Here, we used the fourth scale of age (in days), which provides a relatively uniform distribution of individual ages across lifespan^8^. We then combined the interpolated expression values for each dataset, and used these to run the k-means algorithm. Because the clustering analysis was conducted to study the diversity of turning points, we tried a range of cluster numbers and ensured that different choices of k yield the same conclusion with respect to the diversity of peak expression times (data not shown).

### Functional analysis

We used Gene Ontology (GO)^55^ categories for functional analysis. “GO.db”^56^, “AnnotationDbi”^57^ and “org.Hs.eg.db”^58^ libraries in R were used in order to access the GO database and associated gene annotations (date of retrieval: March 26, 2016). In total; we used (1) 13,392 Biological Process, (2) 3,769 Molecular Function and (3) 1,601 Cellular Component categories containing (1) 15,754, (2) 15,805 and (3) 16,768 of the genes expressed in at least one dataset. We tested enrichment of the reversal pattern keeping the developmental change fixed. For instance, the down-up pattern was compared with down-down genes in each GO category, and an enrichment odds ratio (OR) was calculated for genes in that GO category compared to genes not in that GO category. Likewise, the up-down pattern was compared with the up-up pattern. Next, across all 19 datasets, we searched for consistent over-representation (*i.e*. OR >1 for all datasets) of reversal pattern for each GO BP category. The significance of sharing across datasets was tested using random permutations of individual ages (as described earlier). The schematic representation of the permutation test used for the functional analysis is given in Figure S6. To test the contribution of duplicated GO categories to detected shared significance, we repeated the same analysis after removal of such duplicated GO categories (9666 GO BP categories and 15,754 genes). To summarize the shared GO categories, we used the REVIGO^16^ algorithm, which clusters GO categories based on their semantic similarities, with the options similarity cutoff = 0.7, database = “Homo Sapiens”, semantic similarity measure = “SimRel”. The results were visualized in R using the “treemap” library^59^.

### Regulatory analysis

The “biomaRt” library in R was used to access Ensembl and TarBase^60^ Databases, to retrieve miRNA-target gene associations. In total, 1,078 miRNA with 13,458 target genes were analyzed. For the transcription factor binding site (TFBS) determination (1) +/−2000 base pairs of the transcription start site for each gene was extracted using Ensembl annotations, (2) within these sequences, transcription factor binding sites were predicted using the TRANSFAC database and Match algorithm^61^, (3) for each TFBS, phastCon scores were calculated using UCSC Genome Browser 17-way vertebrate Conserved Element table^62^, and (4) conserved TFBS were defined if 80% or more nucleotides had defined phastCon score and if the average score was 0.6 or more. In total 211 TFBS with 16,594 associated genes were analyzed (data courtesy of Xiling Liu and Haiyang Hu). The over-representation analysis was conducted in the same way as for the functional analysis, keeping the developmental pattern fixed and searching for shared enrichment of the reversal pattern among targets of each miRNA/TF. The significance of the results was tested using random permutations of individual ages.

### Differentiation-related genes

Neuronal differentiation-related genes were determined using data from a human iPSC-derived neuronal differentiation dataset^19^. We applied the Spearman correlation test for three stages of differentiation: iPSC, neurosphere and neuron, and genes showing significant correlation with differentiation stage after multiple test correction (q < 0.05) were considered differentiation-related. First, we tested consistency of expression change directions during neuronal differentiation and postnatal brain development, which was low (median = 48% for down- and median = 44% for up-regulated genes), which might be expected as many neuron development-related genes are down-regulated during the postnatal phase of brain development^8^. We then gauged the significance of the up-down reversal trend among genes up-regulated in neuronal differentiation. For this, we used the reversal proportion, calculated as the number of up-down genes/up-up genes, among genes up-regulated in differentiation (we thus control for possible parallels between differentiation and development). We then compared this observed proportion to those calculated from 1,000 random permutations of individual labels in the aging datasets. To calculate the overall significance across datasets, we compared the number of datasets with more than 50% reversal among genes up-regulated in differentiation with 1,000 random permutations (including all datasets). To test whether genes up-regulated in differentiation show more reversal than other genes, we used a permutation test, permuting the labels for differentiation stages. Thus, we simulated the null hypothesis that there is no effect of differentiation, maintaining the association among genes. Since in each permutation, the numbers of differentiation related genes that pass q < 0.05 will be very few, the reversal proportions calculated with these small numbers will vary greatly. Thus we cannot obtain a reliable null distribution for the reversal proportion. In order to maintain the number of differentiation-related genes the same with the real observation, instead of seeking for significant up-regulation in differentiation permutations, we first sorted the genes according to the effect sizes in permutations and then continued the downstream analysis with the most up-regulated *N* genes that are also up-regulated in postnatal development, *N* being the number of genes significantly up-regulated in real differentiation data and also up-regulated in postnatal development. *N* ranged between 476 and 651 in different age-series datasets.

### AD-related genes

We defined AD-related genes as those consistently increasing or decreasing across all 11 AD subdatasets downloaded from GEO (see Table S8). As previously reported^20^, these shared AD-related expression changes were largely in parallel with expression changes during normal aging, such that in all 19 aging datasets, genes up- (and down-) regulated in AD also tended to be up- (and down-) regulated in aging (Figure S10). We then used the 19 development and 11 AD datasets to test whether genes upregulated in development are downregulated in AD more than by chance. We first found common up-regulated genes (717 genes) among 19 development datasets and common up- and down- regulated genes (690 and 653 genes, respectively) among 11 AD datasets (p < 0.01 for both up and down genes). Then, we calculated the proportion of genes up-regulated in developmental and down-regulated in AD to genes up-regulated in both development and AD, which was 0.73. To test if this observed proportion is significant, we used a permutation test. We performed 1,000 permutations in each AD dataset by randomly mixing AD and control groups and repeating the analysis. As explained in the section above, because calculating proportions from small gene numbers (obtained in the permutations) will introduce noise, we used the following approach: we first sorted the genes by decreasing order in terms of the number of AD datasets in which a gene showed change in same direction. From this sorted list of genes, we chose same number of genes as the observed number of common genes (690 and 653 for up and down respectively) to calculate proportions as above for 1,000 permutations and obtained the null distribution. We then tested the significance of the up-down reversal trend among genes down-regulated in AD. For this, we calculated the reversal proportion (in this case, up-down genes/down-down genes) among genes down-regulated in AD. We thus control for the mentioned parallel changes between aging and AD. We then compared this observed proportion to those calculated from 1,000 random permutations of individual labels in the development datasets. To calculate the overall significance across datasets, we compared the number of datasets with more than 50% reversal among genes down-regulated in AD with 1,000 random permutations (including all datasets).

### Cell type-specific expression analysis

Two different cell type specific expression datasets^17, 18^ were used to analyze relative contribution of different cell types to the expression profile of the samples. Because we have only data available from different platforms, we could not apply sophisticated deconvolution algorithms, such as CIBERSORT^63^, and therefore analyzed the relative contribution of different cell types’ transcriptomes to each sample in the age-series datasets using a simple linear regression-based deconvolution approach. For both datasets, we first calculated the mean expression levels across the replicates of each main cell type: astrocytes (A), oligodendrocytes (Oli), myelinating oligodendrocytes (M_Oli), oligodendrocyte precursor cells (OPC), neurons (N), fetal quiescent (FQuies), fetal replicating (FRep), and endothelial (E). The relative contributions are represented by the regression coefficients calculated according to the following linear regression models:

$$\begin{array}{rcl}{{\rm Z}}_{sample} & = & \alpha +{\beta }_{A}{\zeta }_{A}+{\beta }_{Oli}{\zeta }_{Oli}+{\beta }_{M\_Oli}{\zeta }_{M\_Oli}+{\beta }_{OPC}{\zeta }_{OPC}+{\beta }_{N}{\zeta }_{N}+\varepsilon ,\\  &  & \quad \quad \quad \quad \quad \quad \quad \quad \quad \quad \quad \quad \quad \quad ({\rm{For}}\,{\rm{the}}\,{\rm{Cahoy}}2008\,{\rm{dataset}})\end{array}$$

$$\begin{array}{rcl}{{\rm Z}}_{sample} & = & \alpha +{\beta }_{A}{\zeta }_{A}+{\beta }_{Oli}{\zeta }_{Oli}+{\beta }_{M}{\zeta }_{M}+{\beta }_{FQuies}{\zeta }_{Fquies}+{\beta }_{FRep}{\zeta }_{FRep}\\  &  & +{\beta }_{E}{\zeta }_{E}+{\beta }_{OPC}{\zeta }_{OPC}+{\beta }_{N}{\zeta }_{N}+\varepsilon ,\\  &  & \quad \quad \quad \quad \quad \quad \quad \quad \quad \quad \quad ({\rm{For}}\,{\rm{the}}\,{\rm{Darmanis}}2015\,{\rm{dataset}})\end{array}$$

where *Ζ* represents the expression profile per gene, *β*’s represent the regression coefficients that estimate the relative contributions of each cell type, *ζ*’s represent expression level of each cell type (averaged across replicates), and *ε* represents residuals. The models were fit using the R “lm” function.

### Different turning points

In order to assess the prevalence of reversals seen in other age groups, we repeated the analysis by taking 5, 10, 15, 30, and 40 years of age as turning points (Figure S13). We see that as the turning point increases the correlations between development datasets increase, whereas the opposite trend is observed for correlations between periods, *i.e*. correlation between development and aging. Similarly, we see that the consistency of gene expression changes in development increases as we increase the age of turning point, whereas the same trend is not observed in aging. The consistency starts to decrease after the age of 20. Consistent with this observation, the number of genes showing consistent reversal increases with increasing the age considered as turning point, except the age of 40. That is expected as the consistency of aging-related changes is low when the age of 40 is used as turning point. One important observation is that for all the ages used as turning points, number of consistent up-down genes is higher than that of down-up. Finally, we also wanted to see whether the functional associations of these patterns overlaps. We see that all turning points share the most GO BP groups with the age of 20 among up-down enriched groups. However, among down-up enriched functional groups, this is not observed; also the overall consistency is lower in down-up than in up-down.

### Confirmation of the 25 up-down genes

For postnatal development we used a prefrontal cortex dataset produced using Affymetrix HG-U133P2 arrays, containing 28 human individuals below age 20^64^. About 1/2 of the individuals used in this study overlap with those used in Somel *et al*. 2010 and thus are not independent, but importantly, the data has been generated using Affymetrix 3′ arrays, which are significantly different from the Gene and Exon Arrays used in the main analyses. The dataset was downloaded as a pre-processed “series matrix file” from NCBI GEO^36^ with ID number GSE11512. Among 23 of the 25 genes represented in this dataset, all were up-regulated during postnatal development (Spearman rho ≥0.49). We confirmed the significance of this result by randomly choosing 23 genes from the dataset 10,000 times (p < 0.0001). For aging, we used the GTEx dataset produced by RNA-sequencing. For each of the 13 brain regions, we calculated the proportion of genes among the 25 shared up-down genes, among which expression levels were down-regulated. The median proportion among the 13 datasets was 0.96. Testing the significance of each result using randomization yielded a p-value of <0.0001 for each brain region, except for caudate basal ganglia, where we estimated p = 0.0518 (FDR corrected).

## Electronic supplementary material

Supplementary Information
