# Supplementary material for: Gene expression reversal toward pre-adult levels in the aging human brain and age-related loss of cellular identity
Source: Sci Rep. 2017 Jul 19;7:5894. doi: 10.1038/s41598-017-05927-4 (PMC5517654; doi:10.1038/s41598-017-05927-4)
Supplement: Supplementary file 1 — Supplementary Information [file 41598_2017_5927_MOESM1_ESM.pdf]

Gene expression reversal toward pre-adult levels in the aging human brain and age-related loss of cellular identity

Authors:

Handan Melike Dönertaş, Hamit İzgi, Altuğ Kamacıoğlu, Zhisong He, Philipp Khaitovich, Mehmet Somel

Supplementary figures

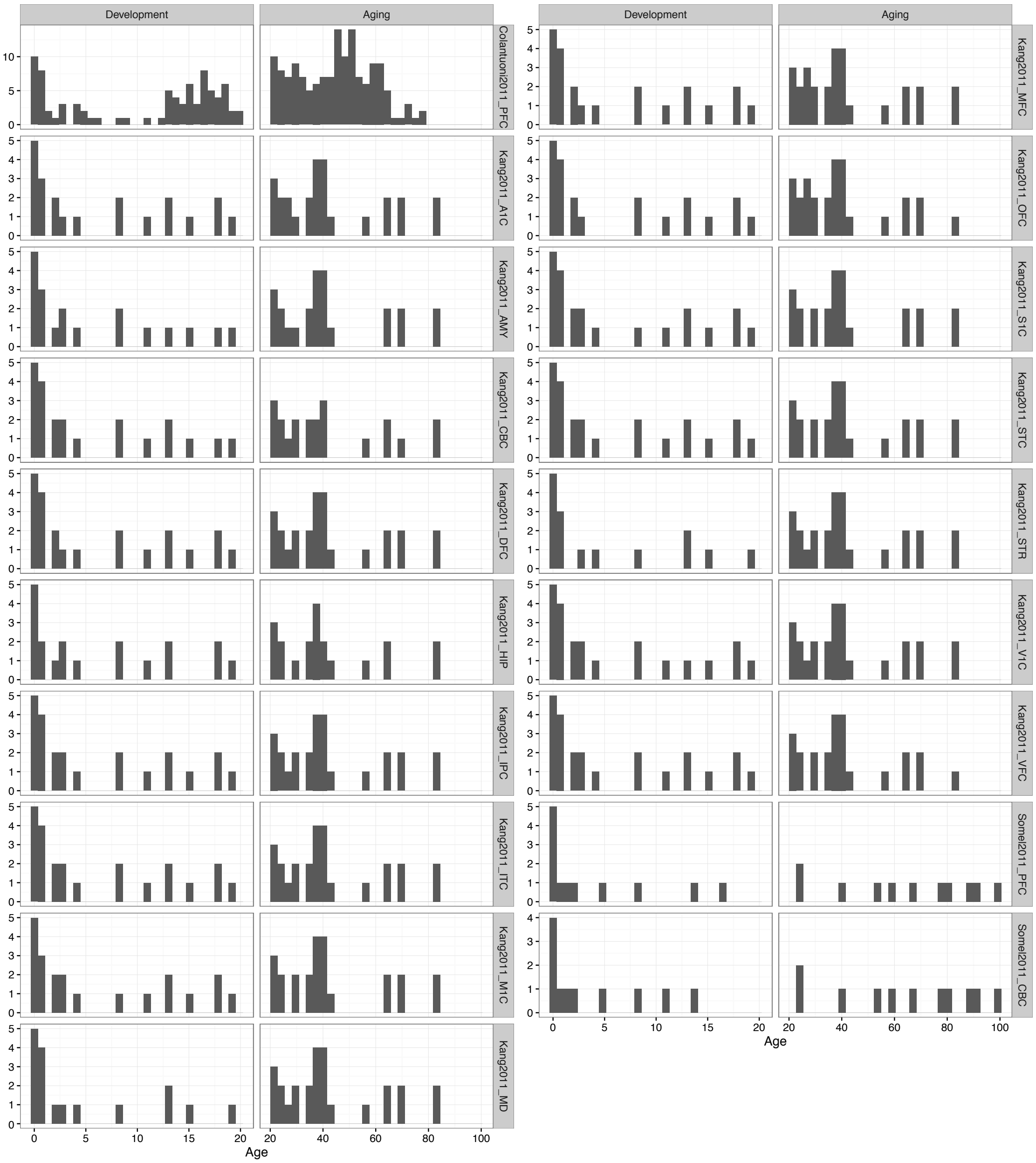

Figure S1: Age distribution of the individuals used in age-series datasets. The y-axis shows the frequency. The dataset name is indicated on the right-hand side.

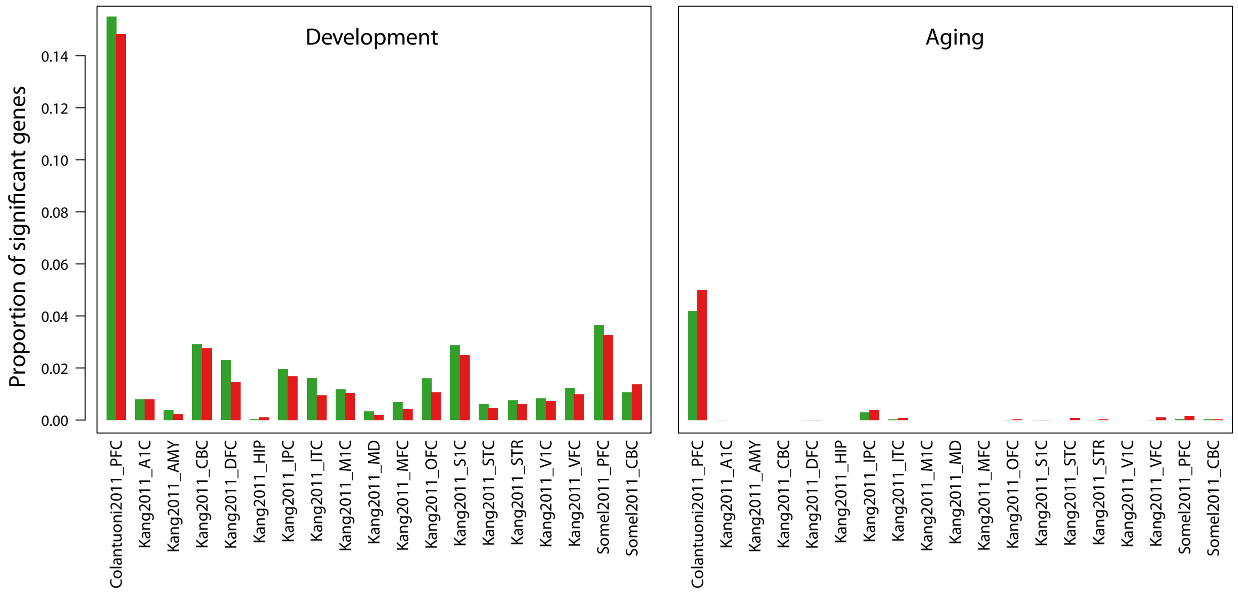

Figure S2: The proportion of the genes with significant expression change (BY corrected Spearman correlation test, q<0.1) within each dataset, calculated separately for development and for aging, and separately for increasing (green) and decreasing (red) genes.

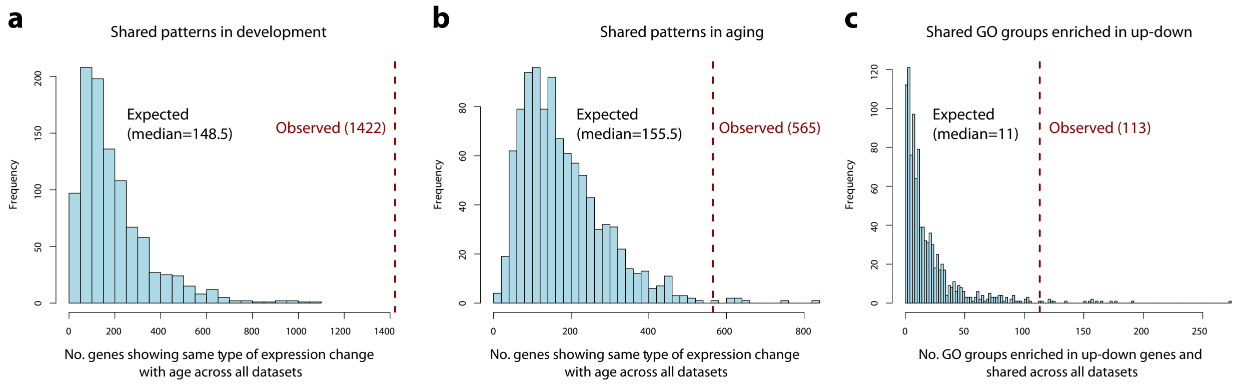

Figure S3: (a) Permutation test result for the number of genes changing in the same direction in development. (b) The same for aging. (c) Permutation test result for the consistency of up-down enriched GO BP categories.

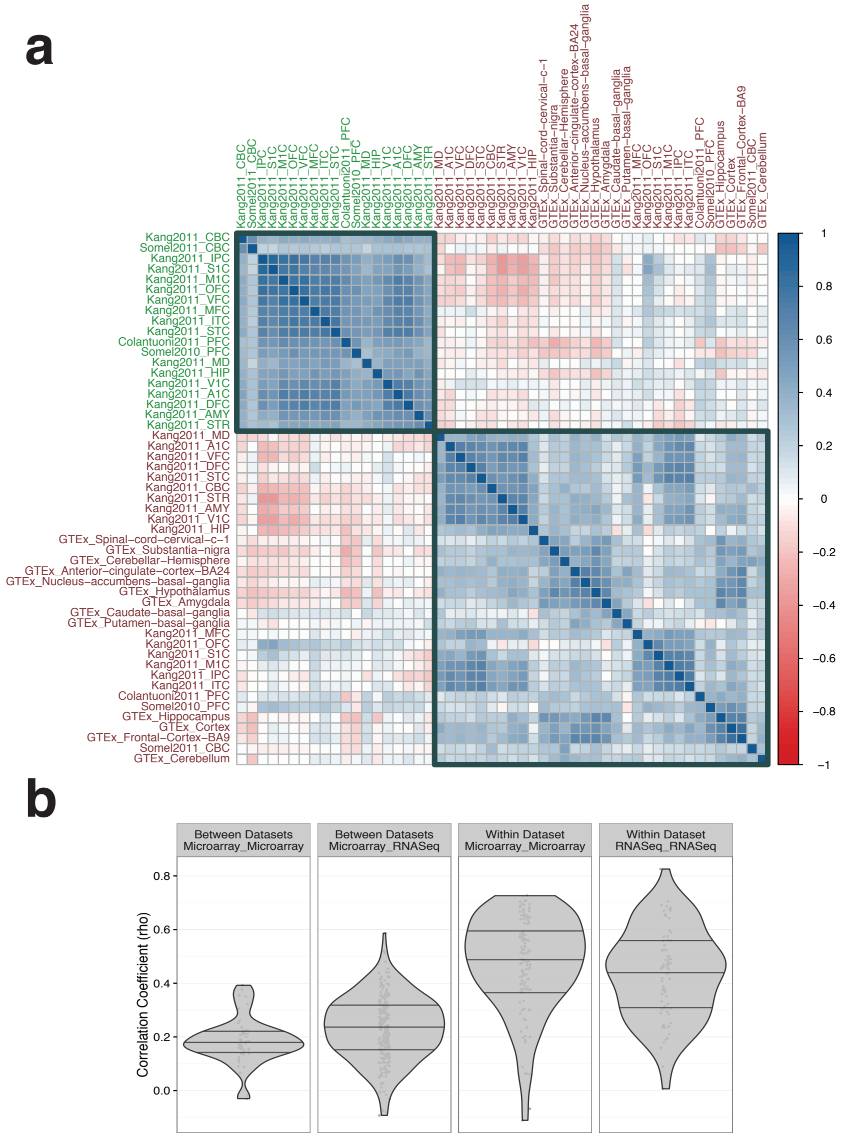

Figure S4: (a) Correlations among expression-age correlation coefficients (in development or in aging) across subdatasets. The color of the squares changes with the magnitude of the Spearman correlation coefficient between pairs of subdatasets; darker colors show stronger negative (red) or positive (blue) correlation. Row and column labels indicate the age period - green: development and, brown: aging. The order of brain regions is determined by agglomerative hierarchical clustering of expression-age correlation coefficients in datasets. (b) Distribution of correlation coefficients between different type of datasets. Between dataset comparisons include pairs of datasets from different studies; e.g. Colantuoni2011_PFC and Kang2011_A1C. Within dataset comparisons include pairs of datasets from the same study; e.g. Kang2011_A1C and Kang2011_AMY. Microarray comparisons include Colantuoni2011, Kang2011 and Somel2011 studies whereas RNASeq data only contains GTEx dataset for different brain regions. It can be seen that being derived from the same platform lead to higher correlations.

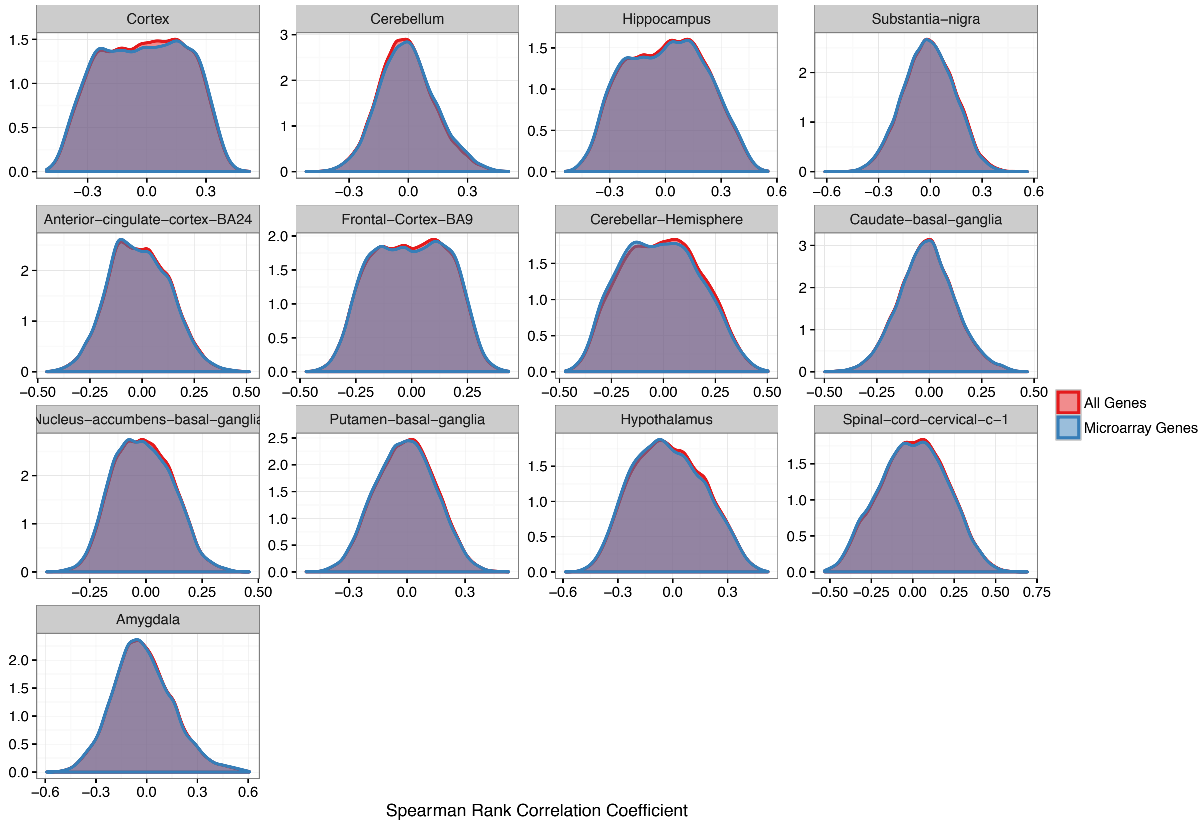

Figure S5: Distribution of aging-related change in different brain regions in GTEx dataset. The x-axis shows the Spearman rank correlation coefficient between expression and age for a gene. The y-axis shows relative frequency calculated by the R "density" function. The colors indicate the group of genes included – red: all genes in a GTEx dataset and, blue: genes in GTEx that are in common with microarray datasets used in the study. The overlap between the two distributions indicates that genes represented on microarrays are not fundamentally different from the rest of the transcriptome with respect to expression changes during brain aging.

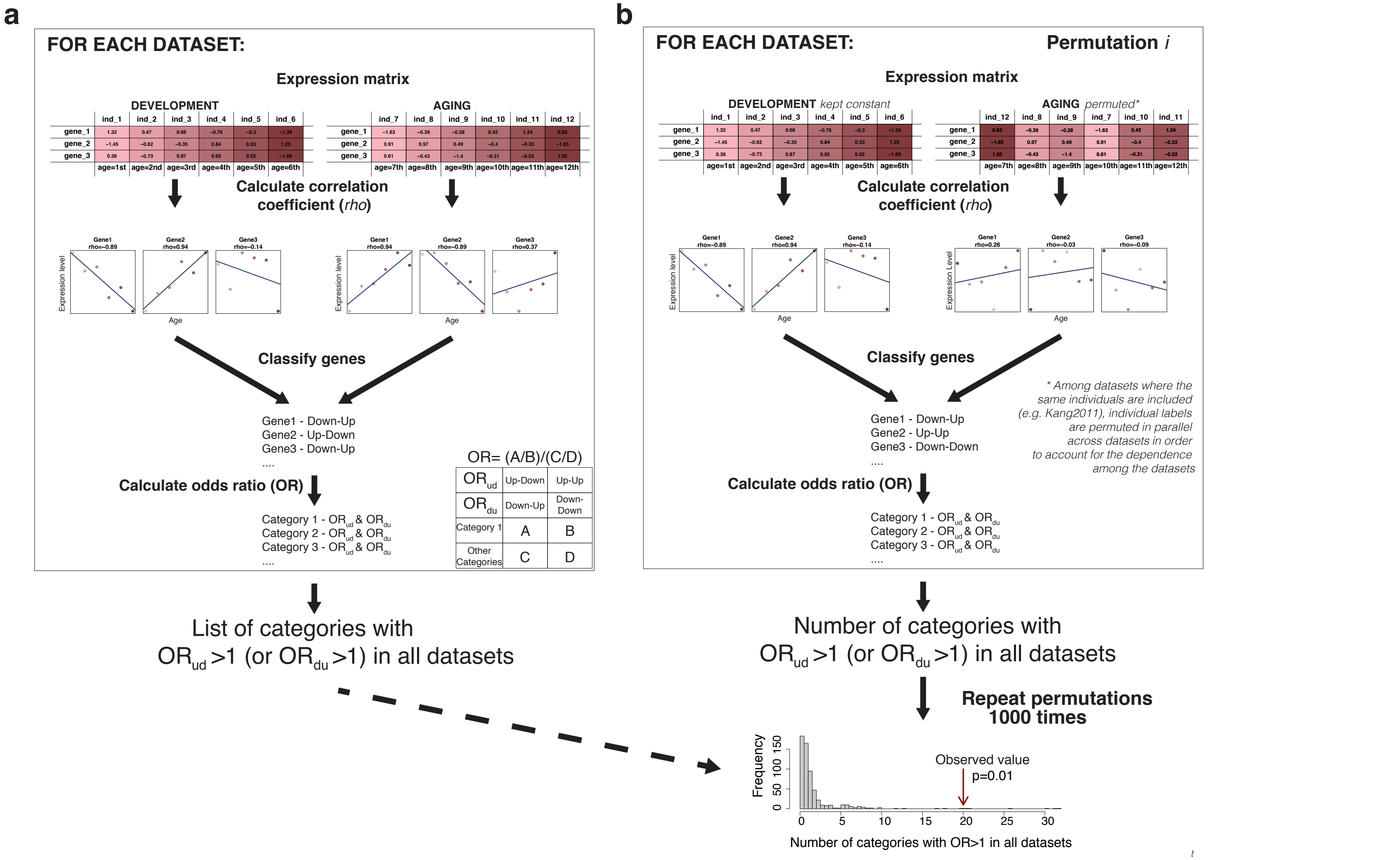

Figure S6: Schematic explanation of the method used for (a) functional analysis and (b) permutation test for functional analysis.

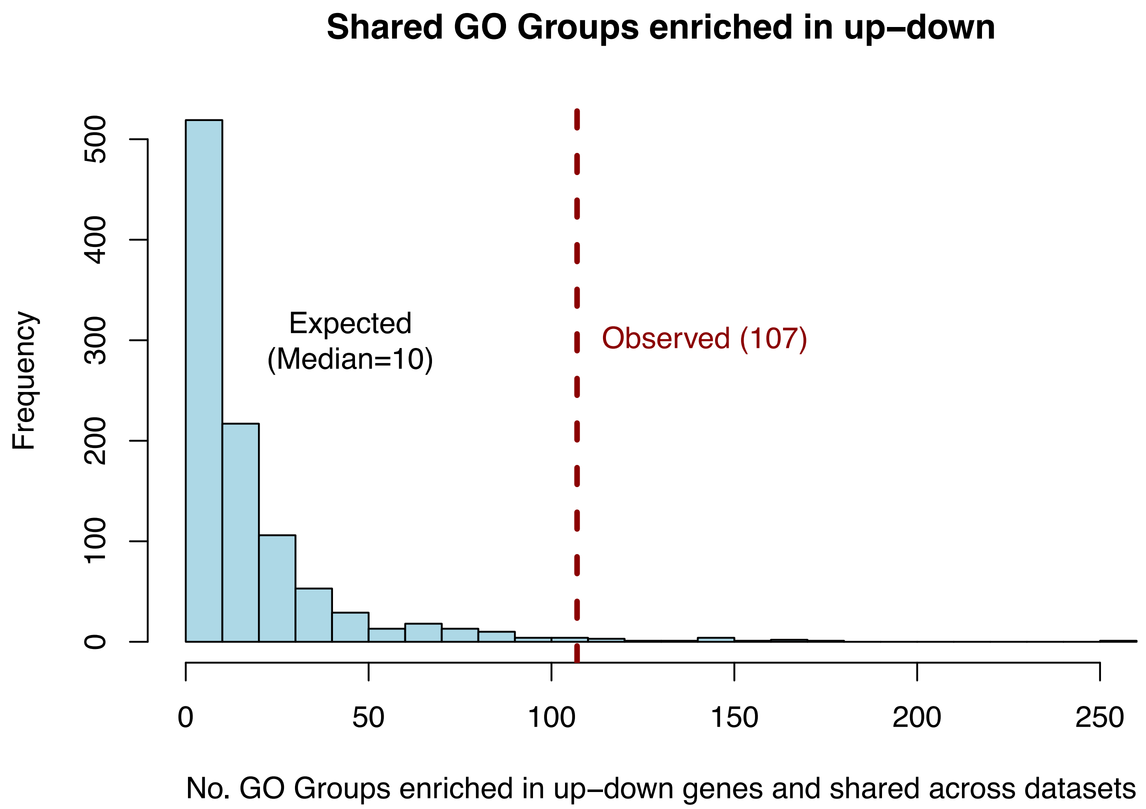

Figure S7: Permutation test result for the consistency of up-down enriched GO BP categories after removal of the duplicate GO groups (permutation test p=0.017, using the same permutations used for generating Figure S3).

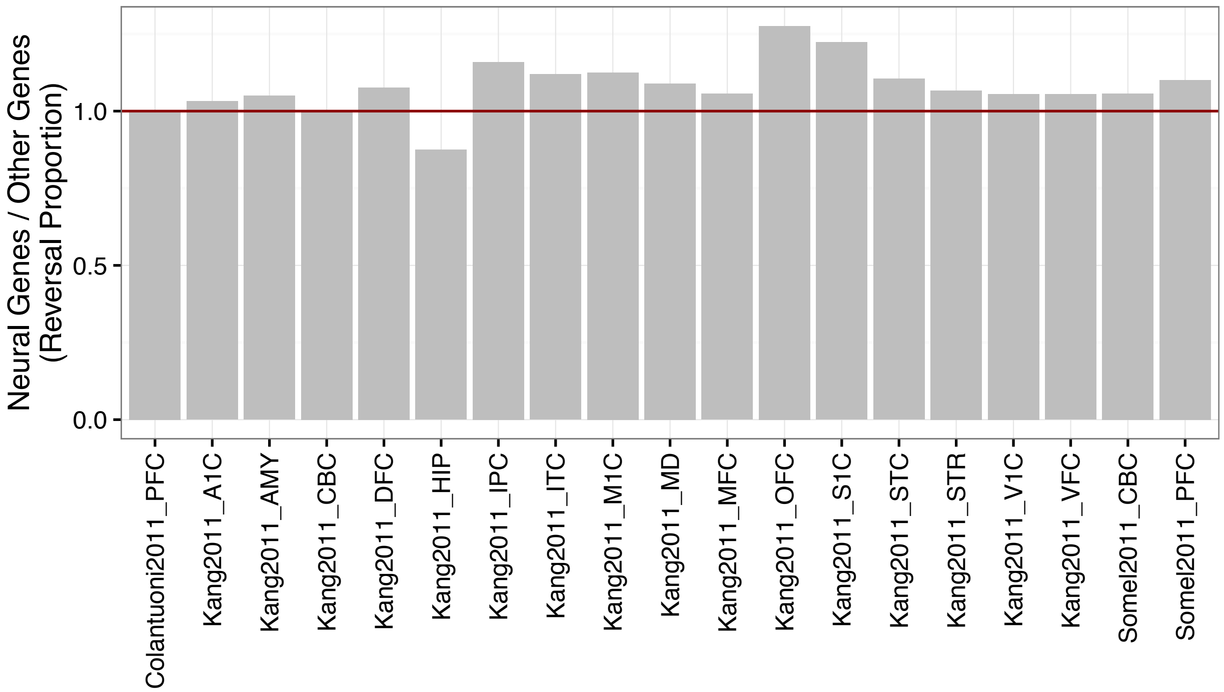

Figure S8: Up-down reversal among all genes assigned to neuron-related categories compared to non-neuronal genes. We joined members of all 581 GO groups that contained the term “neuron” in their definition, which resulted in 3505 genes (overlapping with 2512-2962 genes in each dataset). We then calculated the odds ratio for up-down vs. up-up among neuronal genes compared to all other genes, shown on the y-axis. The odds ratios were >1 among 16/19 datasets (permutation test $p=0.095$).

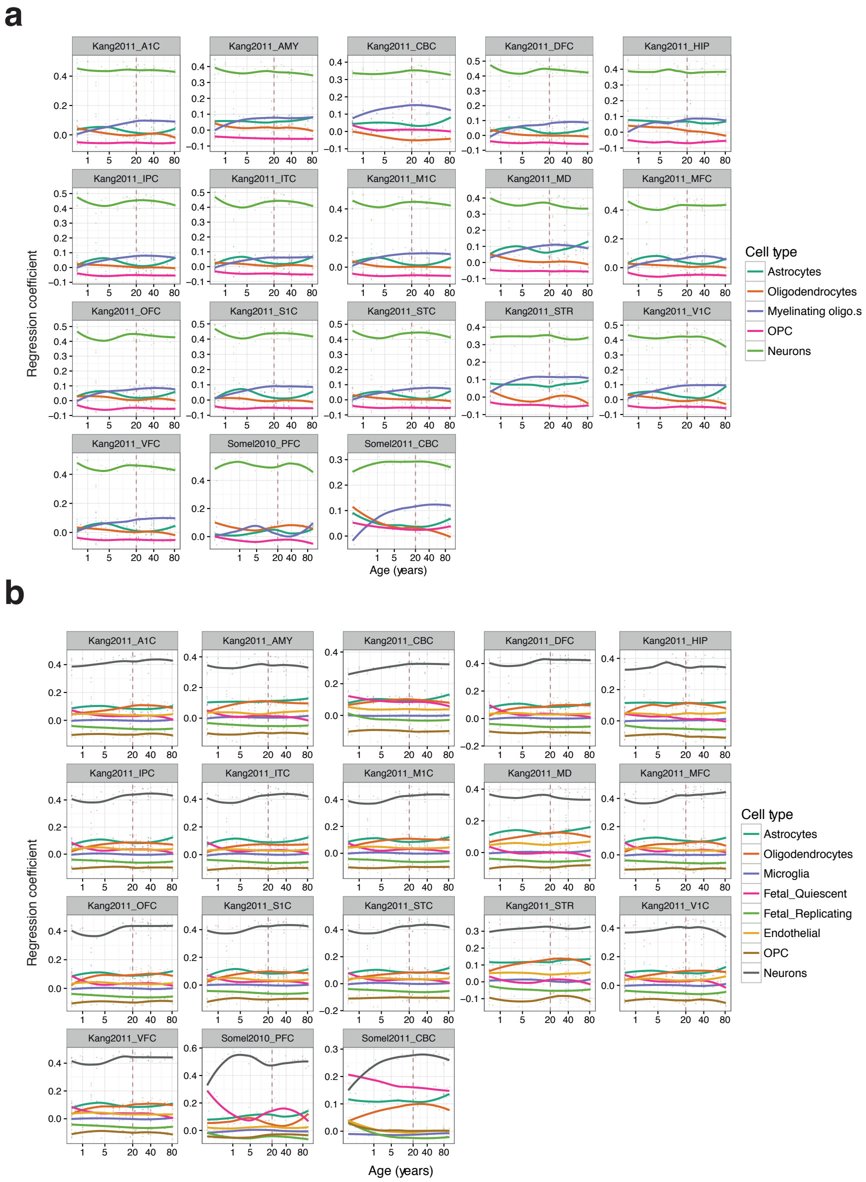

Figure S9: Cell type specific expression analysis using (a) mouse cell-type specific brain microarray and (b) human brain scRNA-seq dataset. x-axis shows individual age on the fourth root scale and y-axis represents the relative contribution of each cell type obtained from the linear model (explained in Methods). The correlations between individual age and neuronal contribution for the aging period ranges between -0.79 and -0.12 (median -0.35) using the mouse cell-type specific brain dataset, and between -0.39 and 0.05 (median -0.10) using the human brain scRNA-seq dataset.

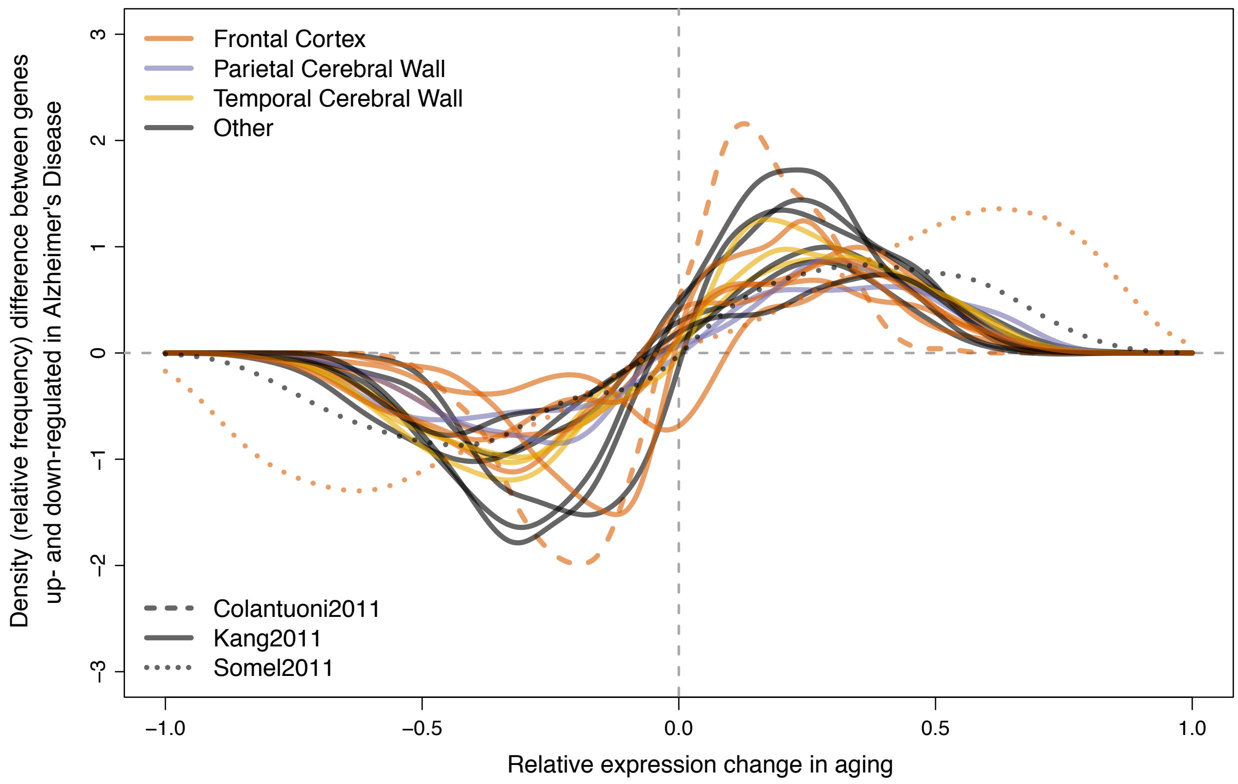

Figure S10: Comparison of gene expression changes in AD brains and in brain aging. Genes were first separated into those up- and down-regulated in Alzheimer's Disease (irrespective of effect size). The y-axis represents difference in the densities (relative frequencies, specifically, the Gaussian kernel density estimate, calculated with the R ''density'' function) of the two gene sets, calculated for relative expression change during aging (x-axis). Positive values on the x-axis indicate up-regulation during aging, and vice versa. Positive (or negative) values on the y-axis indicate an excess (or deficiency) of genes up-regulated in AD at a specific aging-related expression change. Positive y-axis values for genes up-regulated in aging indicate a positive association between AD-related changes and aging-related changes. Different data sources are indicated by different line-types and different brain regions are shown by different colors.

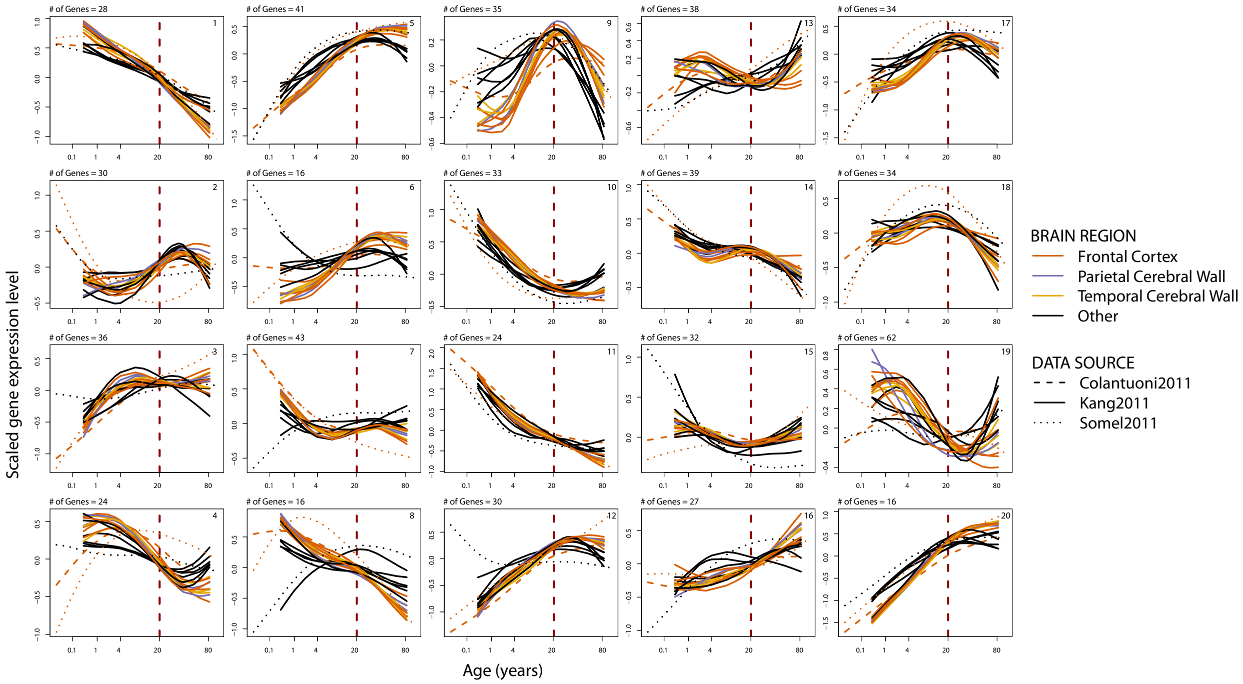

Figure S11: Expression trajectories of the genes associated with synapse-related and up-down enriched GO BP categories. The genes are members of GO BP Group 9 in Figure 3. The y-axis shows the scaled mean expression level of genes in a cluster, and x-axis shows individual age on the fourth root scale. The spline curves were calculated to represent expression change with age among the mean expression levels for a gene cluster, for each dataset and brain region. The cluster numbers are indicated on the upper right corner of each plot.

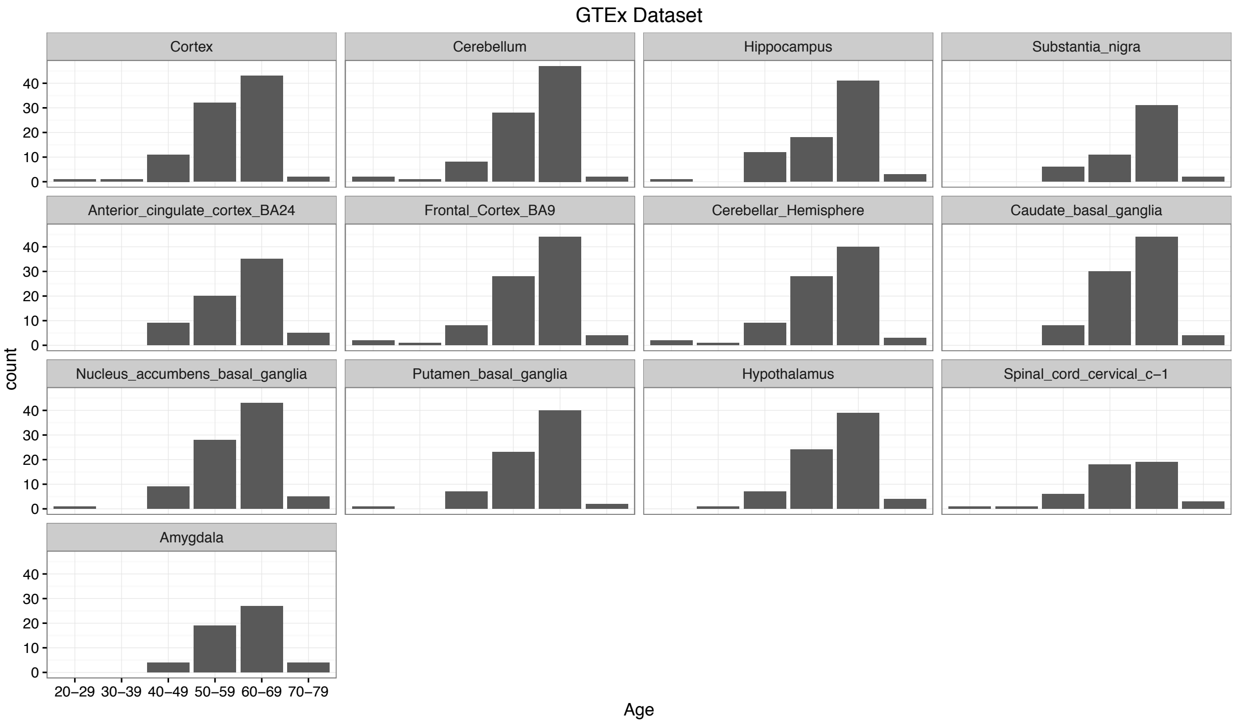

Figure S12: Age distribution of the individuals used in GTEx RNA-Seq datasets.

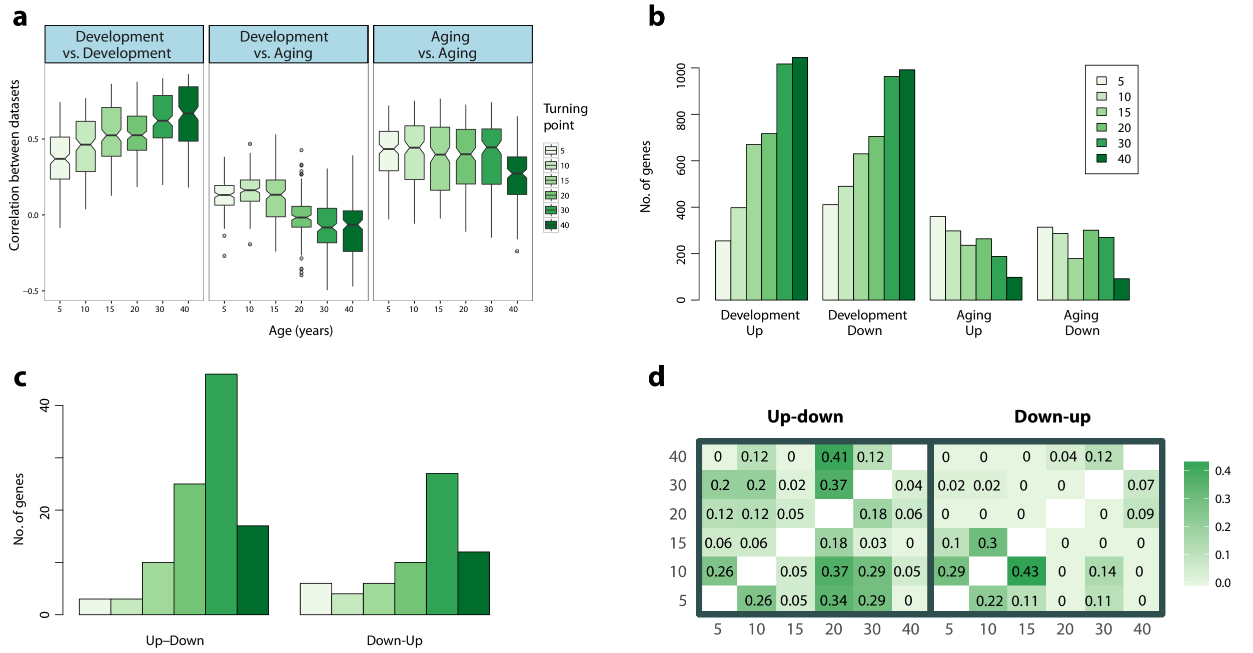

Figure S13: Dividing lifespan at different turning points yields consistent results. (a) Distribution of correlation coefficients between age-related expression changes across datasets (y-axis), identified using different turning points (x-axis). (b) Number of genes showing the same type of change across all datasets (y-axis), i.e. the number of shared genes, identified using different turning points (x-axis). (c) Number of genes showing consistent reversal across all datasets (y-axis), identified using different turning points (x-axis). (d) Proportion of GO BP categories shared when different turning points are used for calculation. The number of categories enriched in up-down or down-up genes (identified using different turning points) and shared between all datasets were calculated, and then compared between each pair of turning points. Each cell indicates the proportion of overlap.

Supplementary tables:

Table S1: Shared reversal genes across all 19 datasets

< 20	> 20	HGNC	Description	Ensembl Gene ID
Up	Down	METTL13	methyltransferase like 13	ENSG00000010165
Up	Down	RNF14	ring finger protein 14	ENSG00000013561
Up	Down	MAPK9	mitogen-activated protein kinase 9	ENSG00000050748
Up	Down	SYT1	synaptotagmin 1	ENSG00000067715
Up	Down	CLCN4	chloride voltage-gated channel 4	ENSG00000073464
Up	Down	SNAP29	synaptosome associated protein 29	ENSG00000099940
Up	Down	SLC25A14	solute carrier family 25 member 14	ENSG00000102078
Up	Down	GRPEL1	GrpE like 1, mitochondrial	ENSG00000109519
Up	Down	CAP2	CAP, adenylate cyclase-associated protein, 2 (yeast)	ENSG00000112186
Up	Down	DNAJC27	DnaJ heat shock protein family (Hsp40) member C27	ENSG00000115137
Up	Down	PPP1R7	protein phosphatase 1 regulatory subunit 7	ENSG00000115685
Up	Down	B4GALT6	beta-1,4-galactosyltransferase 6	ENSG00000118276
Up	Down	FAM98A	family with sequence similarity 98 member A	ENSG00000119812
Up	Down	GOT1	glutamic-oxaloacetic transaminase 1	ENSG00000120053
Up	Down	RNF128	ring finger protein 128, E3 ubiquitin protein ligase	ENSG00000133135
Up	Down	RALB	RAS like proto-oncogene B	ENSG00000144118
Up	Down	MAL2	mal, T-cell differentiation protein 2 (gene/pseudogene)	ENSG00000147676
Up	Down	ELMO1	engulfment and cell motility 1	ENSG00000155849
Up	Down	TRUB1	TruB pseudouridine synthase family member 1	ENSG00000165832
Up	Down	LARP6	La ribonucleoprotein domain family member 6	ENSG00000166173
Up	Down	TRIM44	tripartite motif containing 44	ENSG00000166326
Up	Down	PRNP	prion protein	ENSG00000171867
Up	Down	MAP6D1	MAP6 domain containing 1	ENSG00000180834
Up	Down	AMIGO1	adhesion molecule with Ig like domain 1	ENSG00000181754
Up	Down	NAP1L2	nucleosome assembly protein 1 like 2	ENSG00000186462
Down	Up	IL32	interleukin 32	ENSG00000008517
Down	Up	REV3L	REV3 like, DNA directed polymerase zeta catalytic subunit	ENSG00000009413
Down	Up	SOX9	SRY-box 9	ENSG00000125398
Down	Up	GDPD2	glycerophosphodiester phosphodiesterase domain containing 2	ENSG00000130055
Down	Up	FKBP10	FK506 binding protein 10	ENSG00000141756
Down	Up	ILK	integrin linked kinase	ENSG00000166333
Down	Up	ID4	inhibitor of DNA binding 4, HLH protein	ENSG00000172201
Down	Up	APOLD1	apolipoprotein L domain containing 1	ENSG00000178878
Down	Up	ZNF254	zinc finger protein 254	ENSG00000213096
Down	Up	CCNL2	cyclin L2	ENSG0000221978

Table S2: GO BP Categories enriched in down-up patterns (p=0.4)

GO_ID	GO_TERM
GO:0000060	protein import into nucleus, translocation
GO:0006907	pinocytosis
GO:0010939	regulation of necrotic cell death
GO:0030279	negative regulation of ossification
GO:0032480	negative regulation of type I interferon production
GO:0033574	response to testosterone
GO:0043981	histone H4-K5 acetylation
GO:0043982	histone H4-K8 acetylation
GO:0045601	regulation of endothelial cell differentiation
GO:0072273	metanephric nephron morphogenesis
GO:2000826	regulation of heart morphogenesis

Table S3: GO BP Categories enriched in up-down patterns (p=0.017)

GO_ID	GO_TERM
GO:0000186	activation of MAPKK activity
GO:0000209	protein polyubiquitination
GO:0000266	mitochondrial fission
GO:0000462	maturation of SSU-rRNA from tricistronic rRNA transcript (SSU-rRNA, 5.8S rRNA, LSU-rRNA)
GO:0000902	cell morphogenesis
GO:0000904	cell morphogenesis involved in differentiation
GO:0001963	synaptic transmission, dopaminergic
GO:0001964	startle response
GO:0006464	cellular protein modification process
GO:0006470	protein dephosphorylation
GO:0006511	ubiquitin-dependent protein catabolic process
GO:0006810	transport
GO:0006887	exocytosis
GO:0006904	vesicle docking involved in exocytosis
GO:0006986	response to unfolded protein
GO:0007006	mitochondrial membrane organization
GO:0007158	neuron cell-cell adhesion
GO:0007169	transmembrane receptor protein tyrosine kinase signaling pathway
GO:0007173	epidermal growth factor receptor signaling pathway
GO:0007270	neuron-neuron synaptic transmission
GO:0007399	nervous system development
GO:0007409	axonogenesis
GO:0007411	axon guidance
GO:0007613	memory
GO:0007616	long-term memory
GO:0007626	locomotory behavior
GO:0008038	neuron recognition
GO:0008104	protein localization
GO:0008286	insulin receptor signaling pathway
GO:0008543	fibroblast growth factor receptor signaling pathway
GO:0009101	glycoprotein biosynthetic process
GO:0010469	regulation of receptor activity
GO:0010498	proteasomal protein catabolic process
GO:0015693	magnesium ion transport
GO:0016192	vesicle-mediated transport
GO:0016241	regulation of macroautophagy
GO:0016242	negative regulation of macroautophagy
GO:0019941	modification-dependent protein catabolic process
GO:0022406	membrane docking
GO:0022615	protein to membrane docking
GO:0030111	regulation of Wnt signaling pathway
GO:0030182	neuron differentiation
GO:0030210	heparin biosynthetic process
GO:0030534	adult behavior
GO:0031175	neuron projection development
GO:0031400	negative regulation of protein modification process
GO:0031669	cellular response to nutrient levels
GO:0032229	negative regulation of synaptic transmission, GABAergic
GO:0032989	cellular component morphogenesis

GO:0032990	cell part morphogenesis
GO:0033036	macromolecule localization
GO:0034199	activation of protein kinase A activity
GO:0034613	cellular protein localization
GO:0035249	synaptic transmission, glutamatergic
GO:0036211	protein modification process
GO:0038093	Fc receptor signaling pathway
GO:0038095	Fc-epsilon receptor signaling pathway
GO:0038127	ERBB signaling pathway
GO:0038179	neurotrophin signaling pathway
GO:0042220	response to cocaine
GO:0043161	proteasome-mediated ubiquitin-dependent protein catabolic process
GO:0043266	regulation of potassium ion transport
GO:0043267	negative regulation of potassium ion transport
GO:0043412	macromolecule modification
GO:0043434	response to peptide hormone
GO:0043632	modification-dependent macromolecule catabolic process
GO:0044070	regulation of anion transport
GO:0044267	cellular protein metabolic process
GO:0044344	cellular response to fibroblast growth factor stimulus
GO:0044765	single-organism transport
GO:0045184	establishment of protein localization
GO:0045921	positive regulation of exocytosis
GO:0046888	negative regulation of hormone secretion
GO:0046907	intracellular transport
GO:0048011	neurotrophin TRK receptor signaling pathway
GO:0048278	vesicle docking
GO:0048588	developmental cell growth
GO:0048666	neuron development
GO:0048667	cell morphogenesis involved in neuron differentiation
GO:0048699	generation of neurons
GO:0048812	neuron projection morphogenesis
GO:0048858	cell projection morphogenesis
GO:0050805	negative regulation of synaptic transmission
GO:0050808	synapse organization
GO:0050890	cognition
GO:0050966	detection of mechanical stimulus involved in sensory perception of pain
GO:0051179	localization
GO:0051234	establishment of localization
GO:0051641	cellular localization
GO:0051648	vesicle localization
GO:0051649	establishment of localization in cell
GO:0051650	establishment of vesicle localization
GO:0060341	regulation of cellular localization
GO:0060359	response to ammonium ion
GO:0061564	axon development
GO:0070727	cellular macromolecule localization
GO:0071377	cellular response to glucagon stimulus
GO:0071702	organic substance transport
GO:0071774	response to fibroblast growth factor
GO:0072657	protein localization to membrane
GO:0090140	regulation of mitochondrial fission
GO:0097119	postsynaptic density protein 95 clustering

GO:0097484	dendrite extension
GO:0097485	neuron projection guidance
GO:1901379	regulation of potassium ion transmembrane transport
GO:1901698	response to nitrogen compound
GO:1901699	cellular response to nitrogen compound
GO:1902578	single-organism localization
GO:1902580	single-organism cellular localization
GO:1902591	single-organism membrane budding
GO:1903859	regulation of dendrite extension
GO:1903861	positive regulation of dendrite extension
GO:2000785	regulation of autophagosome assembly

Table S4: GO MF Categories enriched in down-up patterns (p= 0.607)

GO_ID	GO_TERM
GO:0016894	endonuclease activity, active with either ribo- or deoxyribonucleic acids and producing 3'-phosphomonoesters
GO:0030332	cyclin binding

Table S5: GO MF Categories enriched in up-down patterns (p= 0.037)

GO_ID	GO_TERM
GO:0003924	GTPase activity
GO:0004842	ubiquitin-protein transferase activity
GO:0005388	calcium-transporting ATPase activity
GO:0008081	phosphoric diester hydrolase activity
GO:0008171	O-methyltransferase activity
GO:0008194	UDP-glycosyltransferase activity
GO:0008378	galactosyltransferase activity
GO:0017017	MAP kinase tyrosine/serine/threonine phosphatase activity
GO:0019787	ubiquitin-like protein transferase activity
GO:0030515	snoRNA binding
GO:0034235	GPI anchor binding
GO:0050750	low-density lipoprotein particle receptor binding
GO:0051219	phosphoprotein binding
GO:0061630	ubiquitin protein ligase activity
GO:0061659	ubiquitin-like protein ligase activity

Table S6: GO CC Categories enriched in down-up patterns (p= 0.336)

GO_ID	GO_TERM
GO:0002080	acrosomal membrane
GO:0042575	DNA polymerase complex
GO:0072562	blood microparticle

Table S7: GO CC Categories enriched in up-down patterns (p= 0.008)

GO_ID	GO_TERM
GO:0000139	Golgi membrane
GO:0005795	Golgi stack
GO:0008021	synaptic vesicle
GO:0016020	membrane
GO:0016342	catenin complex
GO:0016600	flotillin complex
GO:0030424	axon
GO:0030425	dendrite
GO:0030665	clathrin-coated vesicle membrane
GO:0031985	Golgi cisterna
GO:0034388	Pwp2p-containing subcomplex of 90S preribosome
GO:0036477	somatodendritic compartment
GO:0043025	neuronal cell body
GO:0043198	dendritic shaft
GO:0044297	cell body
GO:0044309	neuron spine
GO:0044431	Golgi apparatus part
GO:0045202	synapse
GO:0098588	bounding membrane of organelle
GO:0098793	presynapse
GO:0098796	membrane protein complex

Table S8: Summary of the datasets used for determination of Alzheimer's Disease-related genes.

GEO accession	Data Source Name	Platform	# of samples	Brain region	#of AD & ND
GSE1297	Blalock2004	HG-U133A	29	HC	20AD-9ND
GSE36980	Hokama2014	HuGene-1_0-st	39	HC	7AD-10ND
				PFC	15AD-18ND
				TC	10AD-19ND
GSE37263	Tan2010	HuEx-1_0-st gene	16	TC	8AD-8ND
GSE39420	Antonell2013	HuGene-1_1-st	14	PC	7AD-7ND
GSE29378	Miller2013	Illumina humanHT- V3.0 beadchip	32	HC	15AD-17ND
GSE33000	Narayanan2014	Rosetta/Merck Human 44k 1.1	466	PFC	310 AD-156ND
GSE44772	Zhang2013	Rosetta/Merck Human 44k 1.1	228	PFC	129AD-99ND
				VCX	129AD-99ND
				CRB	129AD-99ND
